# Li–P–S Electrolyte Materials as a Benchmark for Machine-Learned Interatomic Potentials

**DOI:** 10.1021/acs.jctc.5c02006

**Published:** 2026-03-18

**Authors:** Natascia L. Fragapane, Volker L. Deringer

**Affiliations:** Inorganic Chemistry Laboratory, Department of Chemistry, 6396 University of Oxford, Oxford OX1 3QR, United Kingdom

## Abstract

With the growing availability of machine-learned interatomic potential (MLIP) models for materials simulations, there is an increasing demand for robust, automated, and chemically informed benchmarking methodologies. In response, we here introduce LiPS-25, a curated benchmark data set for a canonical series of solid-state electrolyte materials from the Li_2_S–P_2_S_5_ pseudobinary compositional line, including crystalline and amorphous configurations. Together with the data set, we present a suite of performance tests that range from conventional numerical error metrics to physically motivated evaluation tasks. With a focus on graph-based MLIP architectures, we then show examples of using this data set to conduct numerical experiments, systematically assessing (i) the effect of hyperparameters on task-level performance and (ii) the fine-tuning behavior of selected pretrained (“foundational”) MLIP models. Beyond the Li–P–S solid-state electrolytes, we expect that such benchmarks and accompanying code can be readily adapted to other material systems.

## Graphical abstract

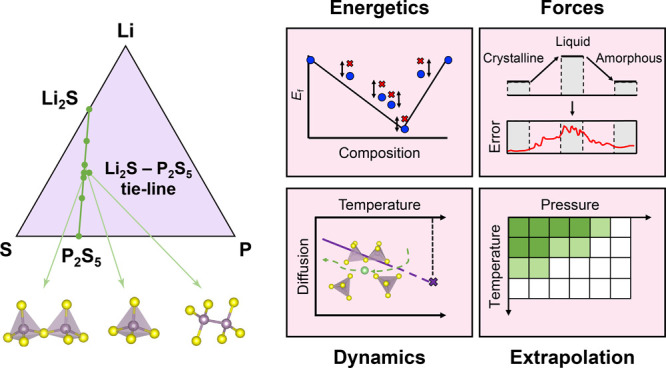

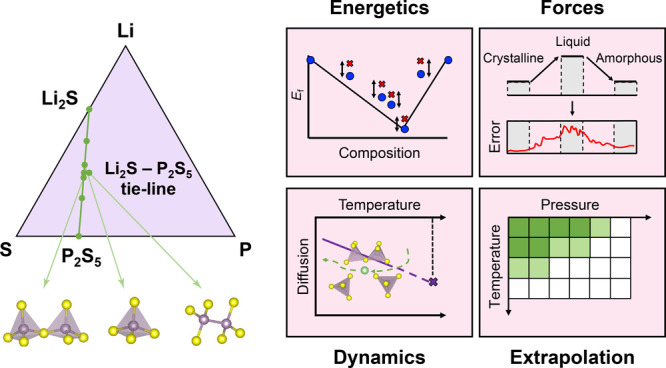

## Introduction

Machine-learned interatomic potentials (MLIPs) are now a standard tool for atomistic simulation, offering first-principles accuracy at a fraction of the computational cost.^1–4^ They have enabled a new degree of realism in materials modeling, with applications including device-scale simulations,^5^ long-time scale dynamics,^6^ and high-throughput materials screening.^7^ More recently, pretrained or “foundation” models^8–17^ have further lowered the barrier to entry: trained on large and diverse data sets, they can be applied to new systems with little to no additional training, dramatically reducing the time and expertise required as compared to crafting MLIPs by hand.

As fitting architectures and specific models continue to proliferate, the systematic and automated benchmarking of MLIPs is becoming ever more important: for identifying state-of-the-art models, clarifying their strengths and limitations, and setting standards for reproducibility and comparison across the field. Several data sets have become widely adopted for evaluating MLIPs.^18–31^ Benchmarks such as QM9,^22^ MD17,^23,30^ and MoleculeNet^25^ have provided insight into model performance on static molecular properties, typically evaluated using established metrics such as the root-mean-square error (RMSE) or mean absolute error (MAE). These metrics, albeit a necessary first step, are not always indicative of accurate model performance in downstream simulations.^32–34^ More physically motivated benchmark tasks have since begun to emerge, for instance in data sets such as OC20/22,^28,29^ frameworks such as MLIPX,^35^ and leaderboard platforms such as Matbench^36,37^ or JARVIS.^38^ And still, comprehensively assessing the robustness and transferability of MLIP models for real-world modeling applications remains a challenge.^39,40^

One such application is the atomistic modeling of lithium thiophosphates (“LiPS” in the following). The LiPS family is a prototypical solid-state electrolyte (SSE) system, combining high ionic conductivity,^41–45^ a wide electrochemical stability range, and low cost.^46^ Materials along the Li_2_S–P_2_S_5_ compositional line (Figure 1a) are of particular interest due to the variety of phases that are accessible depending on preparation conditions: from crystalline to glassy–ceramic and fully amorphous. Li–P–S phases have been the subject of extensive experimental^47–57^ and computational^58–75^ investigation. Their structural complexity makes Li–P–S an informative test system for MLIPs that target this chemical space: a successful model must capture diverse atomic environments and complex dynamic properties arising from them.^76^

**Figure 1:**
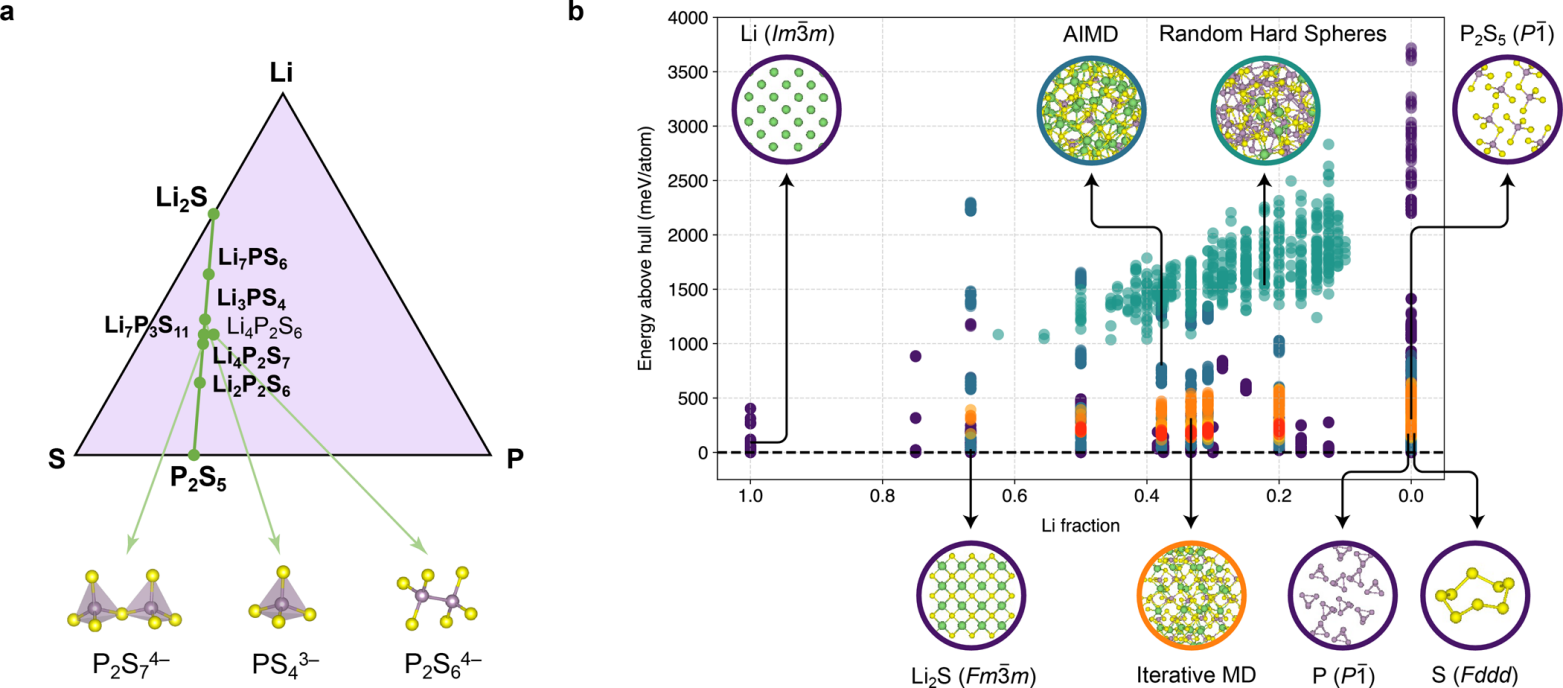
LiPS-25 data set. (a) Ternary diagram of the Li–P–S system, with the tie-line between Li_2_S and P_2_S_5_ indicated, shown in a style similar to ref 66. Green circles mark compositions with known crystalline phases; compositions in bold were used to build the LiPS-25 data set. Key structural motifs are displayed below: *ortho*-thiophosphate, [PS_4_]^3–^; *pyro*-thiophosphate, [P_2_S_7_]^4–^; and *hypo*-thiophosphate, [P_2_S_6_]^4–^. (b) Scatter plot for the LiPS-25 data set showing the fraction of Li in each structure (*x*-axis) versus energy above the convex hull (*y*-axis); a dashed line at *y* = 0 has been added. Dimer configurations are excluded from this plot. Representative structures are shown (atomic color coding: Li, green; P, purple; S, yellow), visualized with VESTA;^82^ colored outlines act as a legend for the scatter plot, with purple for crystalline structures, teal for AIMD snapshots, orange/red for iterative melt–quench configurations (Iter1-*x*, Iter2-*x*), and turquoise for random hard spheres. The arrows are guides to the eye rather than relating to specific datapoints.

Indeed, several recent works have begun exploring benchmarking approaches tailored to Li-ion conductors and SSEs. Therrien et al. introduced a curated data set of SSE materials with experimental ionic conductivities,^77^ while Dembitskiy et al. developed LiTraj, a data set focused on Li-ion migration barriers.^78^ These data sets provide valuable guidance for assessing structure-to-property models and have demonstrated the impact of fine-tuning for foundation models.^78^ A framework introduced by Du et al. broadens the scope of assessment, incorporating properties such as the bulk modulus, energy above the convex hull, and Li-ion diffusion. This methodology enabled a systematic evaluation of pretrained models.^79^ In turn, the recent LiPS20 data set by Bihani et al. compiles a large collection of Li–P–S configurations generated through high-temperature AIMD and augmented with glassy structures.^80^ It provides extensive coverage of high-energy and disordered environments, and evaluation within the EGraFFBench framework includes assessment of RDF agreement and energy and force fidelity throughout annealing trajectories,^80^ allowing for benchmarking of MLIPs on noncrystalline Li–P–S structures. Together, these studies represent important progress toward physically grounded and application-relevant evaluation of ML models for SSEs. They also illustrate the opportunity for data sets of atomistic structures labeled with energies and forces, spanning both crystalline and amorphous structures, that enable comprehensive evaluation of MLIPs on both static and dynamic properties^80^ and support systematic studies of fine-tuning – a strategy shown to be critical for accurately capturing structure and dynamics in glassy SSEs.^81^

Here, we present LiPS-25, a large, first-principles-labeled data set of diverse crystalline and amorphous structures. Following established MLIP training practices, the data set was carefully curated through iterative melt-quench simulations and active learning to comprise representative atomic environments across both ordered and disordered phases: in this way, the data set reflects multiple scenarios relevant for MLIP training. We envisage its utility to be 2-fold: as a general-purpose, off-the-shelf data set for modeling materials across the Li_2_S–P_2_S_5_ tie-line; and as an application-relevant benchmarking tool for MLIPs. To this end, we present a suite of accompanying, physically motivated performance tests, complementing and expanding upon earlier studies,^77–80^ and we report numerical experiments that are underpinned by LiPS-25. Beyond the Li–P–S system, such tests and studies can provide blueprints for future benchmarking efforts in MLIP research and applications.

## The LiPS-25 Data Set

The LiPS-25 data set was curated relying on domain knowledge to cover relevant compositions and polymorphs along the pseudobinary Li_2_S–P_2_S_5_ tie-line, focusing on 7 key compositions (Figure 1) atop a broader coverage of the Li–P–S phase space.

Reference energies and forces (“labels”) were obtained from DFT calculations performed with VASP 6.4.3 ^83–86^ using the PBEsol exchange–correlation functional^87^ and PAW pseudopotentials (PAW_PBE Li_sv 10Sep2004, PAW_PBE P 06Sep2000, and PAW_PBE S 06Sep2000).^88,89^ A plane-wave cutoff of 1000 eV and an SCF energy tolerance of 10^–8^ eV per cell were used, and Brillouin-zone sampling was based on automatically generated *k*-point grids with a maximum spacing of 0.2 Å^–1^. The PBEsol functional was chosen based on previous benchmarking studies for Li_3_PS_4_ (ref 73) and related Li_10_GeP_2_S_12_-type ion conductors.^90^ In ref 90, PBEsol was shown to yield reasonable structural properties, and the predicted lithium diffusion coefficients were found to be largely insensitive to the choice between PBE and PBEsol. In ref 73, PBEsol was found to perform comparably with more computationally expensive alternatives, namely, the meta-GGA r^2^SCAN and the hybrid PBE0 exchange–correlation functionals, in predicting dynamic and phase transition behaviors. While PBE0 more faithfully reproduced electronic band gaps, this is less relevant for the present study, which focuses on atomistic structural and dynamic properties rather than the electronic structure.

The initial data set before iterative training started (which we call “Iter0”) was designed to provide a sufficiently robust starting point for modeling a diverse range of atomic environments, enabling subsequent targeted iterations of data collection. The Iter0 data set consists of several components: distorted and “rattled” elemental, binary, and ternary Li/P/S crystalline structures taken from the Inorganic Crystal Structure Database (ICSD)^91^ and the Materials Project (MP);^92,93^ snapshots from ab initio molecular dynamics (AIMD) simulations at 250, 500, and 1000 K for each of the 7 key compositions considered along the tie-line (Li_2_S, Li_7_PS_6_, Li_3_PS_4_, Li_7_P_3_S_11_, Li_4_P_2_S_7_, Li_2_P_2_S_6_, and P_2_S_5_; Figure 1a); random-hard-sphere structures generated using buildcell;^94^ and isolated dimer configurations of every combination of Li, P, and S atoms.

Iterative melt–quench (MQ) simulations using the NequIP architecture^95^ were then used to extend the data set within an active learning framework. The objective of these iterations (which we call Iter1-*x* and Iter2-*x*, where *x* denotes individual active-learning rounds) is to augment Iter0 by including liquid and amorphous phases, as well as to extend the sampling of disordered crystalline structures.

At each iteration, MQ simulations were performed for the 7 key compositions, and new data were selected from these trajectories using a query-by-committee procedure to select the most “uncertain”, and thus most informative, structures, ensuring a broad coverage of configuration space along the tie-line. Uncertainty was estimated using a committee of five subsampled models, each trained on a random 50% subset of the current data set. These models were used to evaluate snapshots extracted from the MQ trajectories, and the standard deviation of the predicted atomic forces across the committee was used as the uncertainty metric.

The MQ protocols span seven iterations, grouped into Iter1 and Iter2 based on the sampling objective. Iter1-(1–4) formed a more general addition to the data set, with NVT MQ cycles of 300 K → *T*_melt_ → 300 K (*T*_melt_ = 1000, 1500 K; quench rates 50, 100 K/ps); the most uncertain structures across all trajectories were added to the data set. Iter2-(1–3) instead focused on augmenting the data set with glassy structures only. From NPT MQ cycles of 300 K → 1500 K → *T*_quench_ (*T*_quench_ = 300, 400, 500 K; quench rate 50 K/ps), data selection was restricted to the most uncertain structures only within the anneal post melt–quench, thus specifically targeting amorphous structures. Each Iter1/2-*x* iteration corresponds to a complete active-learning cycle of MQ simulation, uncertainty-based selection, DFT labeling, and retraining.

Data set convergence was assessed by monitoring both numerical accuracy on held-out, in-distribution test sets and the reproduction of structural observables for crystalline and amorphous phases. By Iter2-3, improvements in test-set errors had plateaued and key structural metrics, including radial and angular distributions, and the amorphous structure factor,^52^ were well reproduced. On this basis, the data set was deemed converged after a total of seven rounds of iterative data set augmentation, and no further data collection was performed.

The components of the LiPS-25 data set are visualized in Figure 1b and summarized in Table 1, and further details are provided in the Supporting Information. LiPS-25 includes predefined training, validation, and test sets for cross-comparability and consistent model evaluation. The validation set is used to tune hyperparameters and assess model performance during training, whereas the test set is used to evaluate model performance after the training is complete. To ensure that each subset represents the diversity of the complete data set, we employ random stratified sampling with an 80:10:10 split. The data set is openly available at ref 96.

**Table 1:** Composition of the LiPS-25 Data Set^a^

data type	*N*_cells_	*N*_atoms_	*E̅*_hull_ (meV/atom)	*P*_90–10_(*E*_hull_) (meV/atom)
crystalline	8,891	258,880	176	432
AIMD	1,246	103,301	638	1,300
random hard spheres	500	50,553	1,673	673
dimers	138	276	4,840	5,225
Iter1^b^	1,000	52,217	336	325
Iter2^b^	750	66,349	205	74
total	12,525	531,576	348	1,007

a Columns report the number of cells, *N*_cells_, and the total number of atoms, *N*_atoms_, of each structure type, along with the average energy above the convex hull, *E̅*_hull_, and the 10th–90th percentile range of energies above the hull, *P*_90–10_(*E*_hull_).

b “Iter1” and “Iter2”, respectively, refer to all Iter1-*x* and Iter2-*x* data sets combined.

## Benchmark Tasks

To accompany the LiPS-25 data set, we introduce a set of four benchmark tasks (Figure 2) that evaluate MLIP performance on key aspects relevant to SSE modeling. These physically motivated evaluations provide broad yet informative indicators of model suitability, including physical accuracy and dynamic fidelity, that complement static errors. By extending beyond conventional numerical metrics, the benchmarks are able to capture subtle limitations that can affect a model’s applicability to specific materials systems and applications. Each task is designed to balance computational cost with diagnostic value, enabling the systematic comparison of multiple models without the (often prohibitive) computational expense of full-scale production simulations that might demand longer time scales or larger simulation sizes. Crucially, we focus our tasks on physically meaningful observables for which reliable experimental or computational reference data already exist or can be reasonably collected. This focus ensures that the resulting comparisons are both grounded and interpretable within the broader context of SSE research.

**Figure 2:**
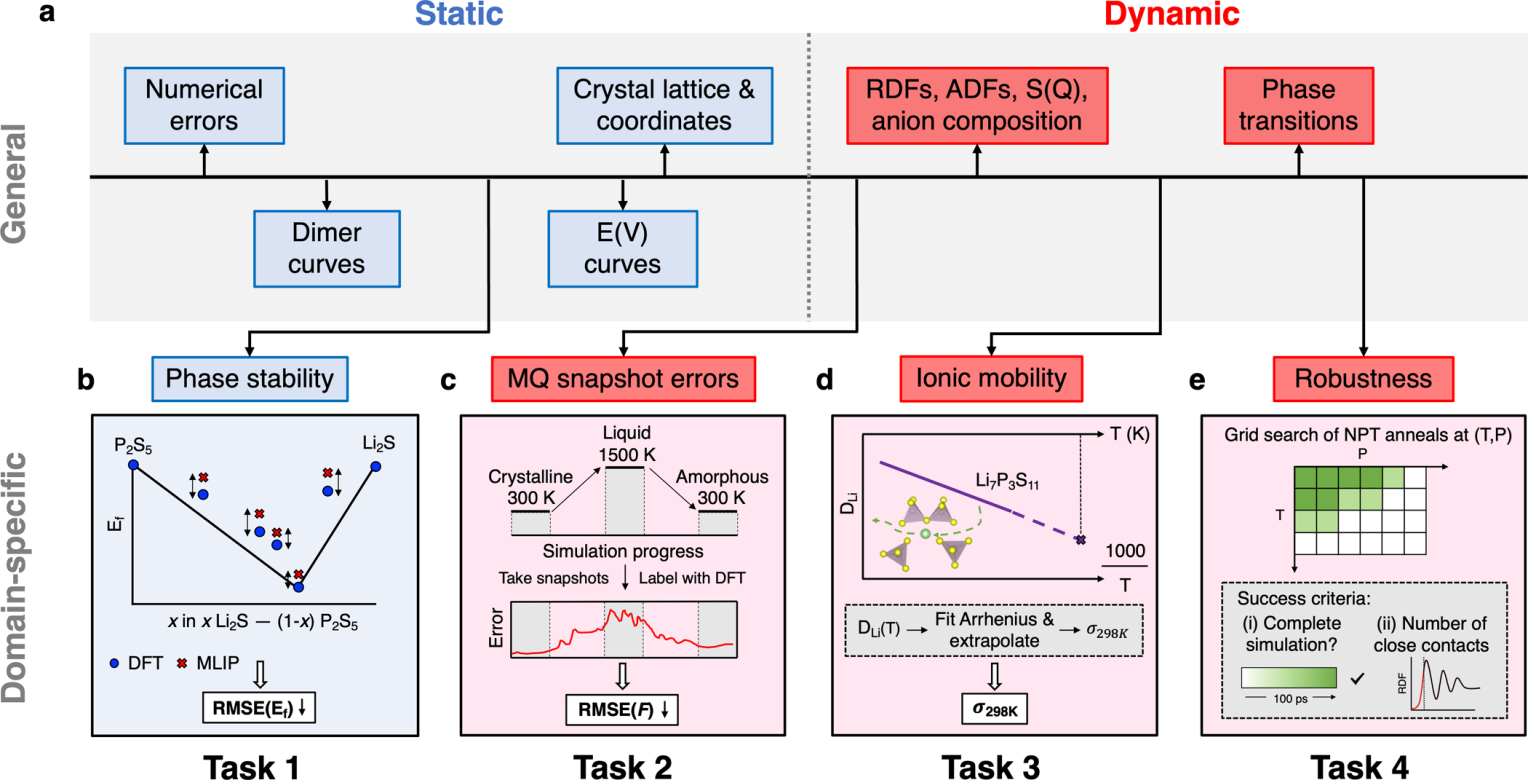
Benchmark tasks. (a) Overview of validation techniques for MLIPs, which fall into two groups: “static” validation, assessing numerical errors and basic energetic profiles, and “dynamic” validation based on MD simulations, leveraging domain expertise to evaluate MLIP performance. From these, four domain-specific benchmarking tasks have been selected to accompany the LiPS-25 data set, facilitating physically motivated evaluation of MLIPs. (b) **Task 1:** Energetic accuracy. The MLIP is used to predict formation energies of 8 crystalline structures along the Li_2_S–P_2_S_5_ tie-line. These predictions are compared against ground-truth values to compute the RMSE­(E_*f*_) metric. (c) **Task 2:** Domain-specific force accuracy. Evenly spaced snapshots are taken from an NPT melt–quench simulation of Li_7_P_3_S_11_. Force errors are calculated with respect to DFT, and aggregated into the RMSE­(*F*) metric. (d) **Task 3:** Property accuracy. The room-temperature ionic conductivity of the Li_7_P_3_S_11_ crystal, a known superionic conductor, is predicted; the inset illustrates Li-ion migration, visualized with VESTA.^82^ (e) **Task 4:** Robustness. NPT simulations across a grid of temperatures and pressures are run and assessed by simulation survival and by the number of close-contact events.

For each task, we describe the aspect of MLIP performance being assessed – such as accuracy on the LiPS-25 data set, generalizability to out-of-domain configurations, or robustness – along with the methodology used and the relevance of the task to SSE modeling. All benchmarks are accompanied by Python notebooks, and, where relevant, LAMMPS input files, to ensure reproducibility and to facilitate the application of these benchmarks to other MLIPs.

### Task 1: Energetic Accuracy

This task assesses the accuracy with which an MLIP reproduces DFT formation energies. In contrast to standard energy MAE/RMSE performance metrics, this task requires both reliable force predictions to obtain correct relaxed geometries and accurate energies to evaluate stability. In this benchmark, formation energies provide a thermodynamic measure of relative stability and energetic ordering of experimentally reported crystalline phases, as well as theoretically proposed phases such as Li_4_P_2_S_7_.^97^ Accurate reproduction of these values ensures that the MLIP faithfully reflects the relative energetic hierarchy among the crystalline structures included in the benchmark.

The formation energy per atom, *E*_f/atom_, is calculated relative to the end-points, Li_2_S and P_2_S_5_, for selected compositions along *x*Li_2_S–(1–*x*)­P_2_S_5_, as in ref 71:

$$E_{\mathrm{f/atom}}=\frac{1}{7-4x}\left[E_{{\left({\mathrm{Li}}_{2}S\right)}_{x}{\left(P_{2}S_{5}\right)}_{1-x}}-xE_{{\mathrm{Li}}_{2}S}-\left(1-x\right)E_{P_{2}S_{5}}\right]$$ 1

*E*_f/atom_ values are obtained from the crystal structures relaxed with the corresponding method (MLIP or DFT), and the RMSE­(*E*_f_) is computed by comparing the MLIP and DFT energy labels. In this method-specific relaxation approach, the RMSE­(*E*_f_) conflates energetic and force accuracy, since models with poor force fidelity may converge to different geometries. To isolate single-point energetic accuracy, we also compute the RMSE­(*E*_f_) from a common set of DFT-relaxed structures. These results are provided in the Supporting Information, and both evaluation protocols are implemented in a Jupyter notebook accompanying the present work.

The eight structures included in this task are Li_2_P_2_S_6_, Li_4_P_2_S_7_, Li_7_P_3_S_11_, α-Li_3_PS_4_, β-Li_3_PS_4_, γ-Li_3_PS_4_, low-temperature Li_7_PS_6_ (*Pna*2_1_), and high-temperature Li_7_PS_6_ (*F*4̅3*m*). Two structures, high-temperature Li_7_PS_6_ and β-Li_3_PS_4_, exhibit intrinsic disorder; in these cases, a single representative configuration was selected for benchmarking (see Supporting Information for structure sources and details on handling partial occupancies). All other structures are fully ordered. We emphasize that for the purposes of this benchmark, the aim is not to account for all possible disordered configurations, but to provide a consistent comparison between the MLIP and DFT.

Of these structures, Li_4_P_2_S_7_, α-Li_3_PS_4_, and high-temperature Li_7_PS_6_ have not been explicitly trained on, and thus present a test for an MLIP’s extrapolation ability.

### Task 2: Domain-Specific Force Accuracy

This task evaluates the accuracy of an MLIP in predicting forces throughout a domain-specific molecular-dynamics (MD) simulation, as in prior work.^98,99^ Here, DFT snapshots were computed every 5 ps from a NequIP-driven melt–quench trajectory of a 672-atom Li_7_P_3_S_11_ supercell between 300 and 1500 K. Force errors were then computed relative to these DFT labels. This test assesses the MLIP’s (i) generalizability, through encompassing the full range of atomic environments relevant to the data set – fully ordered crystalline, through locally ordered amorphous, to highly disordered liquid; and (ii) robustness, as the forces these snapshots experience are directly relevant to MD simulations – thus poor prediction of these forces may indicate future unreliable propagation of dynamics. In contrast to Task 1, which evaluates models on optimized crystal structures only, Task 2 probes the model’s accuracy in nonequilibrium, thermally disordered environments, thereby offering a more demanding and practically relevant evaluation of a given MLIP model.

### Task 3: Property Accuracy

This task evaluates the ability of an MLIP to accurately capture lithium-ion diffusion, a property that emerges from accurate force predictions over extended time scales rather than being an explicit training target. Since lithium-ion mobility is central to SSE performance, models that fail to reproduce diffusion behavior would likely have limited practical value in realistic materials simulations, making this assessment a critical test of model applicability.

In this benchmark, we evaluate the ionic mobility in crystalline Li_7_P_3_S_11_, a widely studied superionic conductor,^42,100,101^ which provides a rich basis for model validation. To minimize the computational expense of this task, we focus on a single representative composition and polymorph, with both the simulation length and box size converged (Figure S2). 500 ps NVT anneals across the relevant temperature regime of 400–800 K are carried out on 672-atom supercells. The diffusion coefficient at 298 K is obtained by an Arrhenius extrapolation, and the corresponding ionic conductivity is estimated from the Nernst–Einstein relation. Full details of the calculation are provided in the Supporting Information.

We note that these approaches carry inherent limitations. Arrhenius extrapolation assumes that the dominant diffusion mechanism remains unchanged and that linear Arrhenius behavior persists from 400–800 K down to room temperature; deviations from these assumptions, as well as uncertainties in the high-temperature diffusion coefficients, can lead to significant errors in extrapolated values. Recent work has shown how such uncertainties may be propagated rigorously using Bayesian methods.^109^ In addition, the Nernst–Einstein relation neglects dynamical correlations between Li-ion charge carriers, and between the carriers and the thiophosphate framework, which can result in an overestimation of the ionic conductivity due to cooperative motion. More accurate alternatives account for correlated motion via charge or center-of-mass diffusion coefficients,^110^ but require substantially longer simulations to achieve sufficient statistical accuracy. For the purposes of this benchmark, however, the Arrhenius and Nernst–Einstein approaches provide a practical and consistently applicable methodology across a large number of MLIP trajectories.

We benchmark the predicted σ_298_ values only by their magnitude, rather than by drawing a direct comparison to specific experimental or computational references, which are compiled in Table 2. This is an intentional design choice – minor variations in experimental synthesis conditions can strongly alter local structural motifs, which can in turn have great influences on the measured ionic conductivity values.^55^ Moreover, such measurements are typically performed on powder samples, where grain boundaries, defects, and amorphous regions play a role – features that no simulation suitable for large-scale and routine benchmarking purposes can fully capture. A detailed discussion of the limitations associated with comparing experimental and computational σ_298_ values is provided in the Supporting Information.

**Table 2:** Room-Temperature Ionic Conductivities (σ_RT_) and Activation Energies (*E*_a_) for Crystalline and Glass-Ceramic Li_7_P_3_S_11_ Reported in the Literature^a^

ref.	method	phase	σ_RT_ (mS/cm)	*E*_a_ (eV)
^42^	solid-state	glass-ceramic	0.08	
^42^	solid-state	glass-ceramic	1.4	0.50
	**solid-state**	**glass-ceramic**	**17** ^b^	**0.17**
^102^	solid-state	glass-ceramic	1.3	0.21
 ^102^	solid-state	glass-ceramic	12	0.18
 ^50^	mechanochemical	glass-ceramic	3.2	0.12
 ^103^	mechanochemical	crystal	4	0.29
 ^104^	mechanochemical	crystal	8.6	0.29
 ^105^	wet chemistry	glass-ceramic	0.27	0.39
 ^106^	wet chemistry	glass-ceramic	0.87	0.37
 ^107^	wet chemistry	glass-ceramic	0.011	
^107^	wet chemistry	glass-ceramic	1.0	0.13
 ^102^	AIMD (PBE)	crystal	57.0	0.19
	**AIMD (PBEsol)**	**crystal**	**61.0** ^c^	
^108^	AIMD (PBE)	crystal	45.7	0.19
 ^63^	AIMD (PBE)	crystal	72.0	0.17
 ^66^	AIMD (PBE)	crystal	84.0	0.17

a Experimental values (top) are compiled based on the review in ref 55, while computational values (bottom) were collected in this work from individual studies. The synthesis or simulation method is indicated for each entry. Multiple values from the same reference reflect differing experimental conditions (e.g., annealing temperatures); full details are given in the original works. An extended table including glass phases is provided in the Supporting Information.

b Highest reported experimental conductivity.^42^

c Representative AIMD value, computed using the same exchange–correlation functional as used for LiPS-25.^102^

Performing a complementary AIMD study at comparable simulation size and time scale would also have been highly expensive, and we refrained from doing so. Instead, Task 3 is intended to evaluate whether an MLIP yields conductivity values within a physically reasonable range. In this way, we consider conductivity values within the range of experiment to AIMD to be acceptable. As points of reference, we highlight in bold in Table 2 the highest reported experimental conductivity of 17 mS/cm,^42^ and a representative AIMD value of 61 mS/cm, computed using the same XC-functional as the DFT labels used in LiPS-25.^102^

### Task 4: Robustness

The final task assesses the robustness of each MLIP. While this test is not specific to SSEs, it provides a general evaluation of the stability and reliability of MD simulations driven by the model, which are essential for any downstream application. Here, we deliberately push the models to extreme conditions as a stress test, in order to probe the limits of their stability and predictive capability; we note that these conditions are far outside those relevant for LiPS systems, and the resulting structures may be nonphysical. We perform 100 ps NPT simulations on a 1008-atom random-hard-sphere structure generated using buildcell,^94^ and relaxed in a fixed cell with the corresponding potential. Simulations are carried out across a grid of temperatures (1000–16,000 K) and pressures (10^6^–10^12^ Pa). Robustness is quantified using two metrics: (i) simulation survival, where a green marker denotes that all three repeats completed 100 ps, pale green indicates partial survival (some, but not all, repeats completed 100 ps), and white denotes complete failure (all three repeats failed); and (ii) the number of close-contact events, defined as the number of frames (sampled at 1 ps intervals) containing interatomic separations ≤1 Å.

## Experiments

### Benchmarking Graph-Based MLIPs

To demonstrate the utility of the tasks accompanying LiPS-25, we study the role of hyperparameters in MACE,^111^ one of the current state-of-the-art architectures for MLIPs. We fit and evaluate a series of 29 models with systematically varying hyperparameters, using the first three tasks introduced above. It should be emphasized that this is not a conventional hyperparameter optimization aimed at minimizing the test-set loss alone, but rather a broader investigation into which model settings yield the most robust, physically meaningful performance in the context of SSE modeling.

We conduct a sweep over four key hyperparameters: (i) the radial cutoff; (ii) the number of message-passing layers; (iii) the number of channels – that is, the multiplicity of node features corresponding to each irreducible representation; (iv) the maximal message equivariance, *L* – that is, the highest degree of the *O*(3) irreducible representations included in the hidden node features of the network. We naively compare hyperparameter values that can be considered to be physically reasonable without requiring detailed domain-knowledge of this system, namely: radial cutoffs between 3–8 Å, 1–5 message-passing layers, 8–256 channels, and equivariance degrees of *L* = 0 (“small”), *L* = 1 (“medium”), and *L* = 2 (“large”). These values correspond to including irreducible representations up to degree *L*, specifically: *L* = 0: 0*e*, *L* = 1: 0*e* + 1*o*, and *L* = 2: 0*e* + 1*o* + 2*e*, where the number denotes the degree *l* of the representation, and the letters *e* and *o* indicate even and odd parity under inversion, respectively.^112^ For further description of the MACE architecture, we direct the reader to refs 111 and 113. All models were trained using the graph-pes software,^114^ with all other hyperparameters set to their graph-pes defaults.

All trained models were sufficiently stable to perform the MD simulations required for Task 3, enabling the calculation of diffusion coefficients. With this baseline established, we next turn to the influence of individual hyperparameters on predictive accuracy, beginning with the radial cutoff. The performance of MACE models with varying radial cutoffs is characterized in Figure 3a. Across all tasks, the smallest radial cutoff of 3 Å is strongly penalized – likely as it fails to capture relevant interactions beyond nearest-neighbor P–S and Li···S pairs that are required for accurate energy and force predictions. While the performance across all three tasks stabilizes from a cutoff of 4 Å, and particularly all Task 3 predictions fall within the expected range of conductivity values according to experiment or previous computations (see Table 2), minor degradations in Task 1 and 2 errors are seen for cutoff radii larger than 6 Å. Hence, a cutoff of 6 Å (indicated in bold in Figure 3) was chosen to be used for subsequent sweeps over layers, channels, and *L* values. MACE models, like most current MLIPs, are inherently local, and designed to capture short- to medium-range interactions; it is plausible that extending the cutoff to include more distant neighbors introduces noise or redundant information, thereby reducing predictive accuracy.

**Figure 3:**
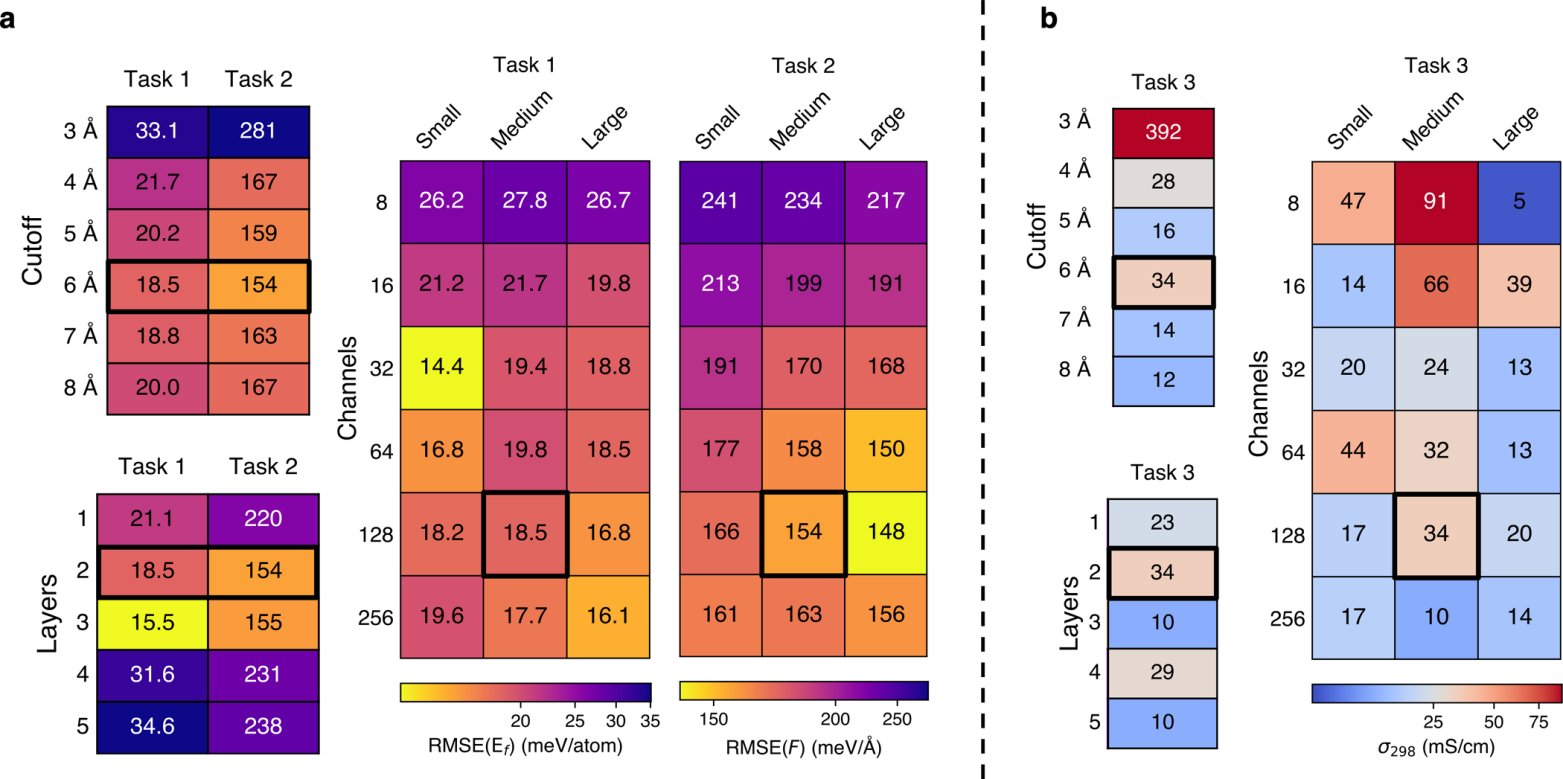
Benchmark task performance for a MACE hyperparameter sweep. (a) Performance for formation energies (**Task 1**) and domain-specific forces (**Task 2**), reported as the mean of five training repeats. Each value reflects the model’s performance under a different hyperparameter configuration, with the metric corresponding to a task-specific prediction error. (b) Performance for the predicted magnitude of ionic conductivity, σ_298_, averaged over three repeats (**Task 3**). As in (a), results reflect variations in model architecture arising from the hyperparameter sweep. In both panels, the boxes outlined in bold indicate the model using a 6 Å cutoff selected from the initial sweep of cutoff radii.

Varying the number of message-passing layers has a pronounced impact on performance for Tasks 1 and 2. Single-layer models are too simplistic to capture the LiPS-25 data set, while deeper architectures (four or five layers) perform worse, possibly due to overfitting or insufficient optimization under the fixed training procedure used here. Two- or three-layer models achieve the best predictive accuracy, suggesting that a moderate depth is expressive enough to capture the system’s complexity while remaining transferable within the LiPS-25 domain. In contrast, Task 3 appears to be less sensitive to the number of layers, with all conductivity predictions remaining within the expected range.

In addition to network depth, the choice of model width – controlled through the number of channels and maximal message equivariance *L* – strongly impacts performance. Our results for Tasks 1 and 2 exhibit clear and consistent trends: narrow models (8–16 channels) systematically underperform, while increasing the number of channels leads to reduced errors up to a point, beyond which the improvements plateau. This suggests the existence of an optimal hyperparameter range that captures the relevant underlying physics without introducing unnecessary model complexity. Notably, in Task 1, which relies on both energy and force accuracy in the crystalline domain, invariant (“small”) models perform competitively, and often outperform their equivariant (“medium”/“large”) counterparts. In contrast, our tests for Task 2, which probes force accuracy across diverse environments, demonstrate the advantages of equivariant architectures for predicting vector quantities such as atomic forces. These models benefit from directly learning force vectors via rotation-aware message passing, whereas invariant models must approximate forces through the gradient of a scalar energy field – an approach that becomes increasingly inaccurate in steep-gradient regimes, such as those encountered in these melt–quench trajectories. The trends for Task 3 are less conclusive: while smaller models produce conductivity estimates that approach the limits of physical plausibility, all models with at least 32 channels yield values within the expected range. However, performance beyond this threshold varies without a consistent trend, underscoring the sensitivity of conductivity predictions to architectural choices. This variability highlights the broader challenge of obtaining reliable and transferable conductivity estimates from simulation alone.

As a final and complementary benchmarking study, we investigate the robustness of one MACE model trained from scratch – specifically the 6 Å cutoff variant highlighted in bold in Figure 3 – and two foundation models, MACE-MP-0b3 and MACE-OMAT-0, using Task 4. The results are shown in Figure 4. Robustness is quantified using two metrics: (i) the survival of the simulation to 100 ps, and (ii) the number of close-contact events, defined as interatomic separations ≤1 Å.

**Figure 4:**
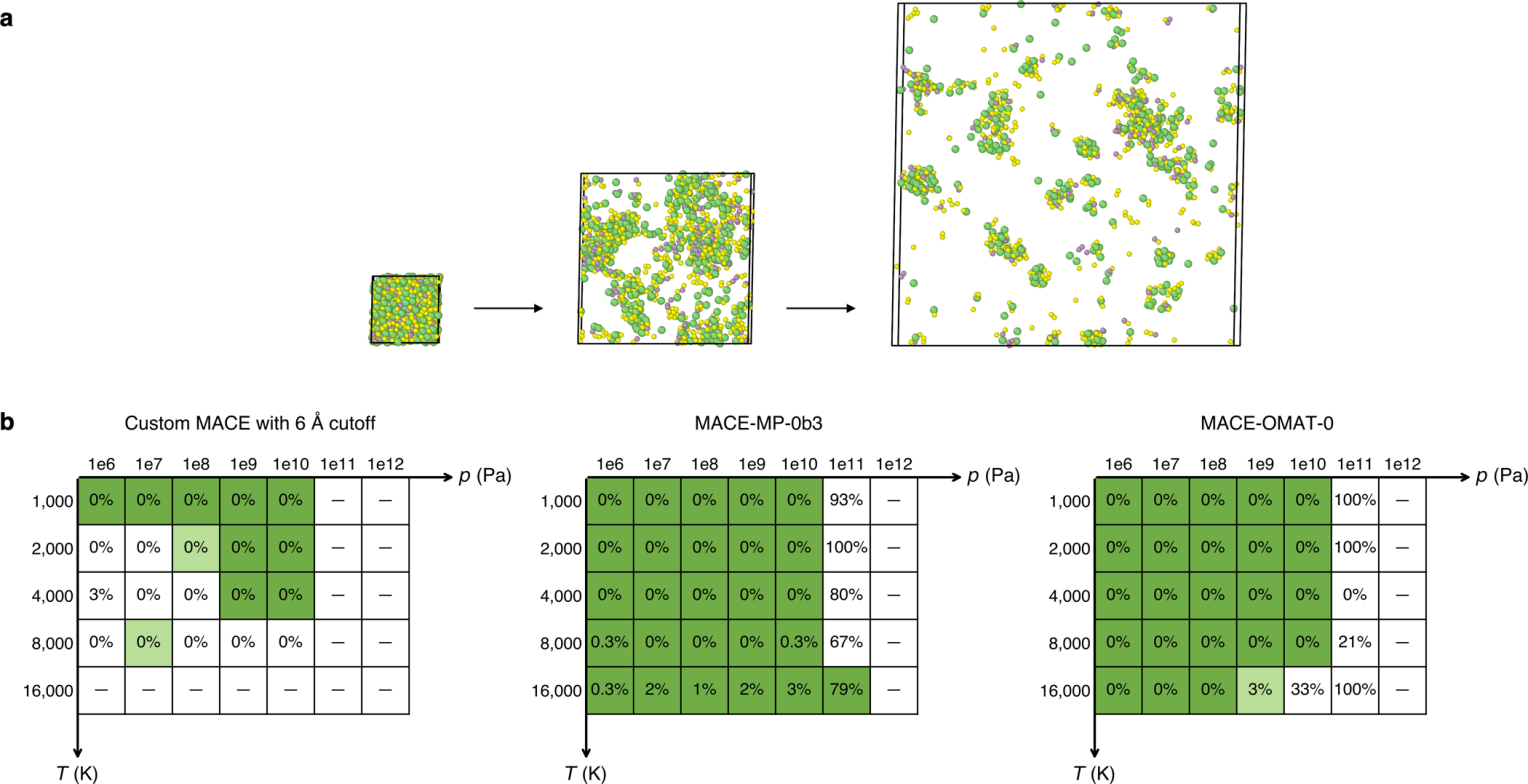
Robustness evaluation of representative MLIP models (**Task 4**). (a) Example trajectory of an NPT annealing simulation, showing the initial relaxed random-hard-sphere structure and two subsequent configurations illustrating expansion and vaporization under high-temperature/-pressure conditions (atomic color coding: Li, green; P, purple; S, yellow). All structures are drawn to scale and were visualized with OVITO.^115^ (b) Grid-search results for three exemplar models: a from-scratch MACE model trained with a 6 Åradial cutoff (from the hyperparameter sweep in Figure 3), and the foundation models MACE-MP-0b3 and MACE-OMAT-0. For each model, 100 ps NPT anneals were performed across a grid of temperatures (1000–16,000 K) and pressures (10^6^–10^12^ Pa) with three repeats. Boxes are shaded green if all repeats reached 100 ps, pale green if one or two repeats reached 100 ps, and white if all repeats failed. The percentages inside the boxes denote the fraction of frames (sampled every 1 ps) with interatomic separations of ≤1 Å. A dash (“–”) indicates that all three repeats failed before 1 ps, i.e., no frames were available for evaluation.

Both foundation models clearly outperform the from-scratch MACE model across these criteria. This is expected, since LiPS-25 is a comparatively narrow training domain, comprising only ∼13k structures sampled up to 2000 K and at ambient pressure (1 atm) during melt–quench iterations. In this sense, the from-scratch model is in fact demonstrating robustness beyond its training domain, with successful simulations observed at temperatures up to 4000 K and pressures up to 10^10^ Pa. In contrast, the foundation models are trained on vastly larger data sets (1.6 M structures for MACE-MP-0b3 and 101 M structures for MACE-OMAT-0), which encompass a broader range of chemical and structural environments.

Interestingly, MACE-MP-0b3 appears robust over a slightly wider range of (*T*, *p*) combinations than MACE-OMAT-0, despite being trained on a data set that is 2 orders of magnitude smaller. While the OMAT data set^116^ was specifically designed to extend the Alexandria data set^117^ with additional off-equilibrium configurations, this larger and more diverse data set does not appear to provide significant stability benefits in extreme temperature–pressure conditions within this specific task.

The close-contact metric proves to be well correlated with simulation survival. For both the from-scratch MACE model and MACE-OMAT-0, simulations that fully survive (marked in green, indicating all three repeats reached 100 ps) invariably contain 0% close-contact frames, whereas partial-survival (in pale green) and failure (in white) regions show progressively higher fractions of such frames. MACE-MP-0b3, however, deviates from this trend: some fully surviving trajectories, such as that at 16,000 K and 10^11^ Pa, contain extremely high fractions of “bad” frames (up to 79%), yet do not fail catastrophically. Taken together, these results demonstrate that while pretraining on large data sets can enhance the stability of MLIPs under extreme conditions, the reliability of the resulting trajectories is not guaranteed and depends on the specific model and simulation regime.

### Fine-Tuning Foundation Models

We further demonstrate the utility of the LiPS-25 data set by using it to fine-tune atomistic foundation models (FMs). To focus this study, we assess the performance of models fine-tuned specifically for the Li_7_P_3_S_11_ composition. A subset of approximately 400 Li_7_P_3_S_11_ structures was extracted from the full LiPS-25 data set to serve as a fine-tuning data set. Several current leading FMs, selected based on their performance on benchmarks such as the Matbench leaderboard at the time of experiment design,^37^ were fine-tuned using graph-pes. We aim to better understand the effects of both the pretraining data set and the model architecture on fine-tuning procedures. We first compare versions of MACE FMs^11^ (namely, MACE-MP-0b3, MACE-MPA-0, MACE-OMAT-0, and MACE-MATPES-PBE-0), which share largely the same architectures and therefore primarily reflect differences in the pretraining data set. We then extend this comparison to the MatterSim^12^ (both MatterSim-1m and MatterSim-5m) and Orb^16,17^ (namely, Orb-v2, Orb-v3-direct-inf-mpa, Orb-v3-direct-inf-omat) families, where architectural differences also play a significant role. Their performance is evaluated in an extended version of Task 2, incorporating both force and energy accuracy tests, as well as on Task 3. To account for differences in reference atomic energies between the pretraining data sets and LiPS-25 arising from variations in exchange–correlation functionals and pseudopotentials, the add_auto_offset feature of graph-pes was applied to correct the zero-shot energy predictions with an offset, thereby aligning them with the energy scale of LiPS-25.

Figure 5 compares several fine-tuned FMs from the MACE, MatterSim, and Orb families. All models shown have been fine-tuned on the same 25 structures, randomly selected from the filtered Li_7_P_3_S_11_ data set; preliminary investigations demonstrated that performance saturates beyond 25 fine-tuning structures (Figure S3a). Across all FMs, it is evident that fine-tuning has a stronger effect on energy errors than force errors (despite having precorrected for effects of different DFT functionals). A possible explanation could be that while fine-tuning shifts the relative positions of minima on the potential-energy surface, it maintains relatively similar gradients between them.

**Figure 5:**
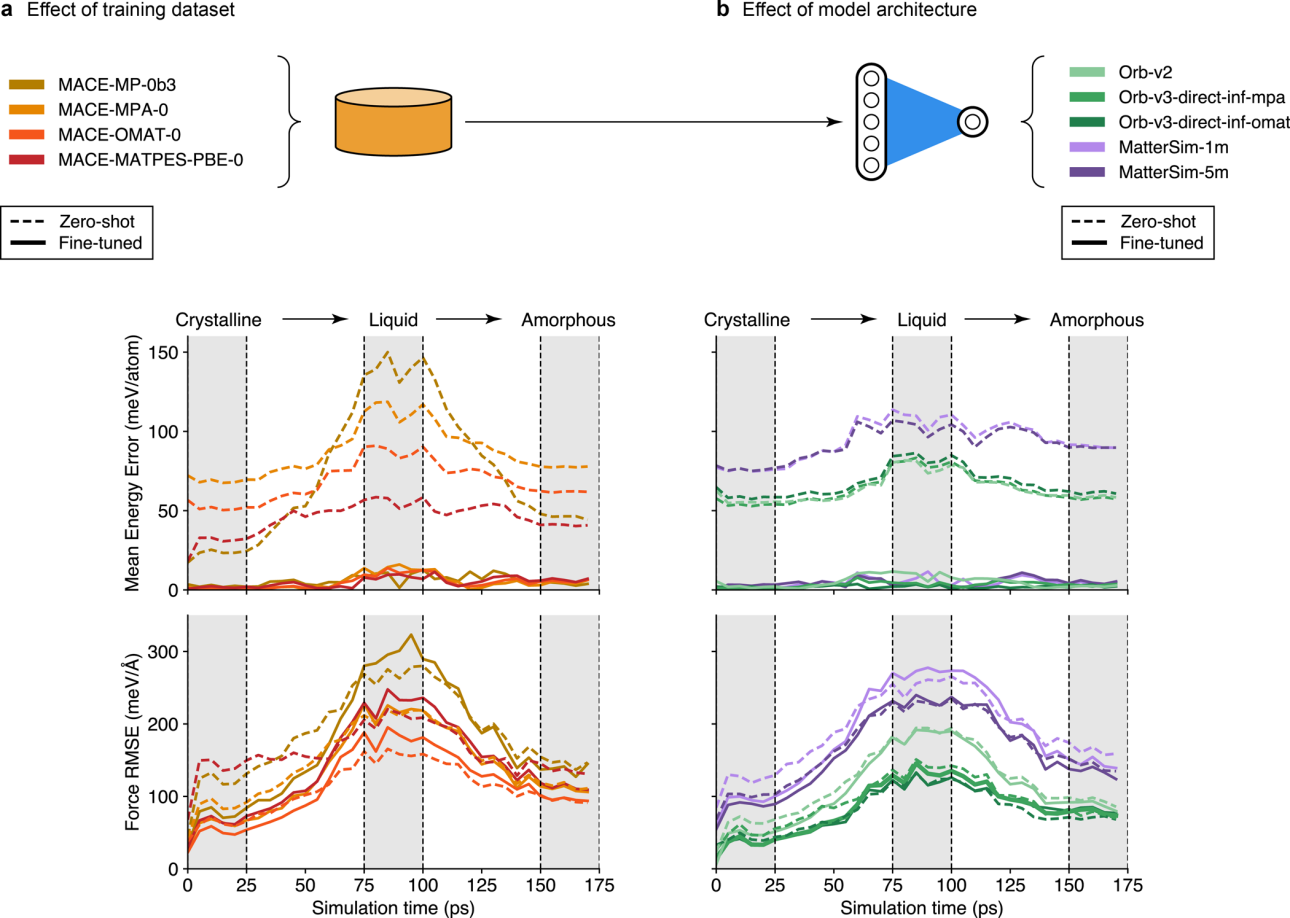
Accuracy of foundation models fine-tuned on 25 Li_7_P_3_S_11_ structures from LiPS-25. We assess these models on a Li_7_P_3_S_11_ melt–quench trajectory (Task 2), showing energy (*top*) and force (*bottom*) errors against DFT-labeled snapshots. Errors for fine-tuned models (solid lines) are averaged over 5 models fine-tuned with different seeds. Zero-shot errors (dashed lines), corresponding to models evaluated without fine-tuning, are shown for comparison. For these zero-shot models, energy predictions were corrected using the add_auto_offset feature of graph-pes to account for differences in reference atomic energies between the pretraining data sets and LiPS-25, arising from the use of different exchange–correlation functionals and pseudopotentials. (a) Performance of MACE foundation models: assessing the effect of differences in training data set for similar architecture. (b) Performance of other foundation model families, viz. MatterSim and Orb: assessing differences in model architecture. The schematics in the upper part are drawn in a style similar to ref 124.

Clear trends emerge in model performance with respect to the choice of pretraining data set. Both the MACE and Orb families exhibit consistent improvements in energy and force errors as the pretraining data set progresses from MPTrj^10^ to MPA^117^ to OMat24 ^116^ – although this effect is notably more pronounced for MACE models. The MPA data set introduces additional structural diversity through the inclusion of the sAlex data set, complementing the DFT-relaxed frames of the MPTrj baseline. This enables models such as MACE-MPA-0 to improve upon their MPTrj-pretrained counterparts, like MACE-MP-0b3. Models pretrained on OMat24 consistently perform best across both the MACE and Orb families. We attribute this advantage to OMat24’s emphasis on nonequilibrium structures, generated by applying perturbations, such as rattling and AIMD, to configurations from the Alexandria data set. We think that this proves especially beneficial in out-of-equilibrium regimes, such as the liquid state explored here, where the difference between OMat24 and other data sets becomes more pronounced. Notably, the MATPES data set,^118^ comprising 400k frames from MD trajectories of MP structures, yields a MACE model with performance comparable to models trained on much larger data sets (1.6 M MPTrj, 12 M MPA, 101 M OMAT24). This underscores the value of judiciously sampled data, and suggests that impactful model development remains feasible even with more modest computational resources.

The MatterSim models were pretrained on a distinct data set comprising structures from the MP, Alexandria, the ICSD, and internally generated configurations.^12^ While increased architectural complexity (from 1m to the 5m version) improves their performance, these models still exhibit higher errors in this task relative to most MACE and all Orb FMs assessed here. This may reflect their comparatively smaller size – even the largest 5M-parameter MatterSim model is notably smaller than other pretrained models such as MACE-MPA-0 (9 M parameters) and Orb-v3 (25 M parameters) – or other architectural differences between frameworks. Further analysis would be required to clarify the respective roles of model capacity and architecture.

The most accurate model overall in this benchmark is Orb-v3-direct-inf-omat, which benefits both from pretraining on the high-quality OMat24 data set and from architectural advantages shared across the Orb family. In particular, its use of direct force prediction, rather than inferring forces from energy gradients, likely contributes to its higher accuracy. However, the numerical gains and efficiency boost of such nonconservative models must be weighed against drawbacks such as poorly converged optimizations and inaccuracies in MD, particularly for collective processes involving long-range correlations, as recently discussed by Bigi et al. (ref 119). These limitations may particularly influence σ_298_ predictions in the present study.

Perhaps most striking, rather than individual model performance, is the collective domain-specific performance of fine-tuned FMs. Within the crystalline regime, fine-tuned FMs can improve force errors upon their zero-shot counterparts by up to a factor of 2. For amorphous structures, the benefit is smaller but often still observable. However, in the liquid state, the fine-tuned models are consistently outperformed by their zero-shot analogues, despite the fact that the fine-tuning data set includes liquid Li_7_P_3_S_11_ structures. This suggests that fine-tuning on a mixed-domain data set can result in loss of knowledge in cases where structural disorder is pronounced. Notably, MACE-MP-0b3 exhibits catastrophic forgetting in the liquid regime, meaning that fine-tuning causes the model to lose previously learned behavior, as evidenced by a marked change in its error profile compared with the zero-shot model. In contrast, the other fine-tuned models retain the same error profile shape as their zero-shot counterparts, albeit with systematically higher errors. Orb models are an exception here – models either reproduce zero-shot behavior or offer small improvements in the liquid regime. These findings highlight a central challenge: fine-tuning can have markedly different effects on model performance in regimes of different degrees of structural disorder, with improvements in one regime sometimes coming at the expense of another. Future work should therefore focus on designing fine-tuning procedures that preserve foundation models’ generalizability and cross-domain robustness, which is essential for realizing their advantages over conventional task-specific models.

### Ionic Conductivities from Fine-Tuned Models

As a final assessment of the fine-tuned FMs, we now proceed to Task 3 to evaluate their performance in predicting the room-temperature ionic conductivity of Li_7_P_3_S_11_. While all zero-shot models exhibit qualitatively correct linear Arrhenius behavior (left-hand-side panels of Figure 6a,b), the corresponding extrapolated ionic conductivities at 298 K vary significantly between models, even by more than an order of magnitude within the same model family (see MACE models in Table 3). Notably, models such as MACE-MP-0 and MACE-MATPES-PBE-0 overpredict ionic conductivity relative to the expected range (see Table 2), consistent with a systematic softening of the underlying PES. This behavior has previously been attributed to pretraining data sets biased toward near-equilibrium configurations, typically derived from DFT relaxation trajectories.^120^ In contrast, models pretrained on more structurally diverse data sets, such as MPA or OMat24, consistently produce zero-shot conductivity predictions within a physically reasonable range, regardless of architecture. This mirrors the trends observed in the domain-specific errors of Task 2 (Figure 5), where the same models exhibited lower errors in high-temperature configurations along the melt–quench trajectory. Such results suggest that strong performance in domain-specific error benchmarks, as in Task 2, can be a useful indicator of reasonable dynamic performance. MatterSim models, on the other hand, tend to underpredict conductivity, which could indicate an overly rigid PES that suppresses ion mobility; here, both pretraining coverage and architectural differences likely contribute to the observed trends.

**Figure 6:**
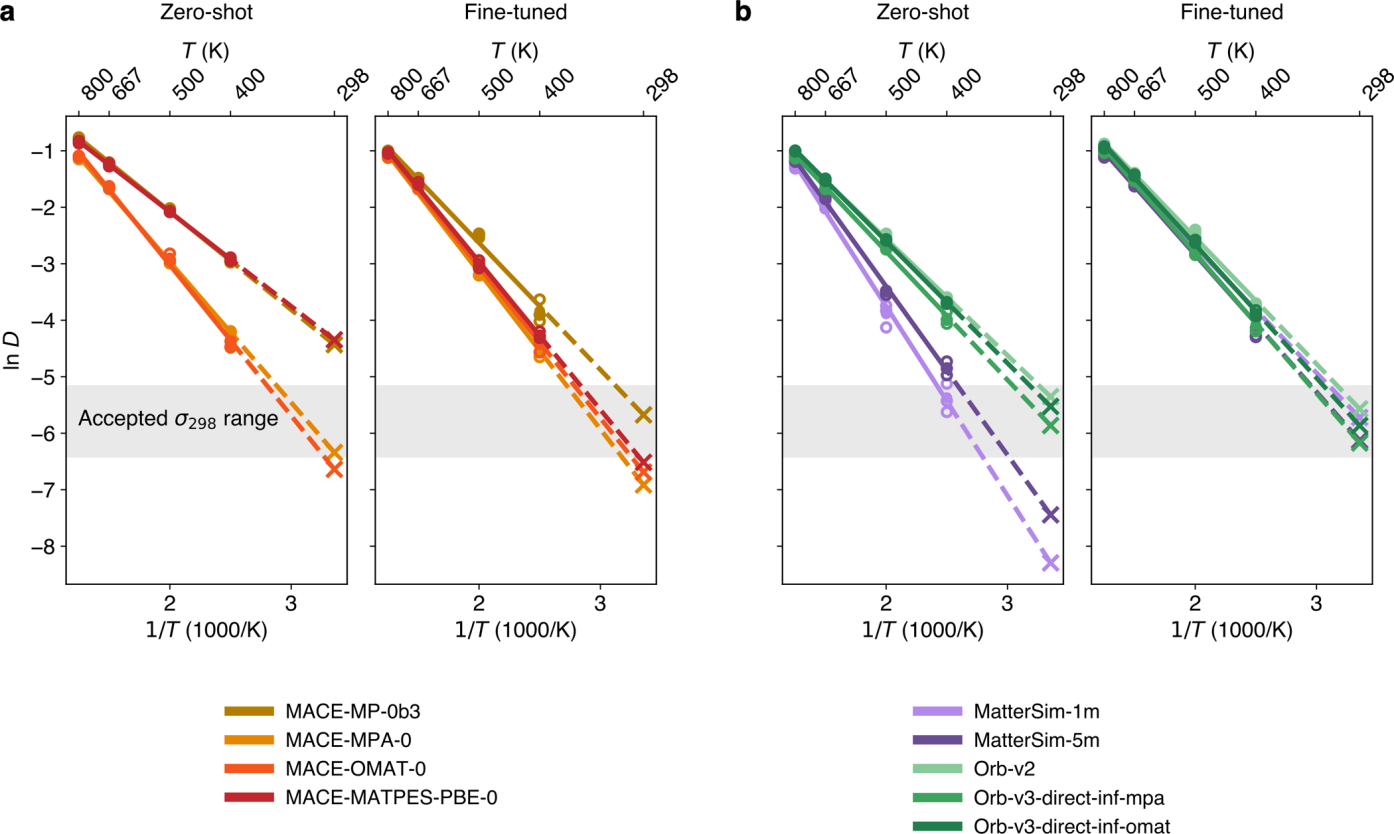
Effect of fine-tuning on room-temperature ionic conductivity predictions for crystalline Li_7_P_3_S_11_. (a) Arrhenius plots for MACE foundation models. (b) Same for for MatterSim and Orb models. In both cases, zero-shot predictions (*left*) are compared to predictions by models that have been fine-tuned on 25 randomly selected Li_7_P_3_S_11_ structures from the LiPS-25 data set (*right*). Hollow circles represent *D*(*T*) values from individual trajectories; filled circles indicate mean *D*(*T*) across three repeats. Solid lines show the best fit to the mean values; dotted lines represent extrapolation to *D*(298 K). The gray shaded region indicates the region of accepted σ_298_ values according to the literature, as described in the main text.

**Table 3:** Predicted Ionic Conductivities of Li_7_P_3_S_11_ at 298 K before and after Fine-Tuning^a^

	σ_298_ (mS/cm)	
model	zero-shot	fine-tuned
MACE-MP-0b3	125	36
MACE-MPA-0	18	11
MACE-OMAT-0	14	13
MACE-MATPES-PBE-0	137	16
MatterSim-1m	3	35
MatterSim-5m	6	23
Orb-v2	50	40
Orb-v3-inf-direct-mpa	30	22
Orb-v3-direct-inf-omat	42	30

a The reported σ_298_ values are calculated from the average diffusion coefficients, *D*(*T*), obtained across three independent repeats (corresponding to the filled-circle data and fitted lines in Figure 6). All values are rounded to the nearest integer.

Fine-tuning the models on the same subset of 25 Li_7_P_3_S_11_ structures as in Figure 5 brings their predictions into much closer agreement, both across and within model families. This convergence is evident in the right-hand-side panels of [Fig fig6]a,b, where the Arrhenius lines of fine-tuned models align more closely. Moreover, all fine-tuned models yield conductivity values within the expected range (see Table 2). Nonetheless, systematic differences between families persist: for example, fine-tuned MACE models consistently predict lower ionic conductivity values than their MatterSim and Orb counterparts (with the exception of MACE-MP-0b3), despite improved overall agreement. These findings highlight the importance of fine-tuning in correcting systematic biases inherited from pretraining, while also suggesting that residual architectural or training differences between model families continue to influence dynamic properties such as ionic conductivity.

Looking ahead, such validation of MLIPs designed for complex functional materials on the physical properties they aim to reproduce should become standard practice. For ionic conductors, this involves assessing transport behavior like ionic conductivity, despite the associated conceptual and practical challenges. Since conductivity predictions are computationally demanding, it is useful to first apply more affordable, targeted tests, such as the domain-specific error analysis as in Task 2, which can serve as strong indicators of dynamic performance. The lack of a clear ground truth complicates validation further: experimental values are not directly comparable to simulations, and generating fully converged AIMD references for each target system would be impractical. As such, future benchmarking efforts should integrate property-level evaluations with complementary analyses, such as inspecting diffusion mechanisms or jump statistics, to ensure that predicted transport arises from physically reasonable processes, even in the absence of an exact conductivity reference.

## Conclusions and Outlook

As machine-learning acceleration becomes the norm in computational materials chemistry, the careful and systematic evaluation of MLIP models is ever more important. The Li–P–S system is well-suited for this purpose: both because of the inherent interest in the materials themselves, and because it represents a broader class of complex chemistries and dynamic phenomena with relevance to battery research. Our LiPS-25 data set supports the fitting of MLIPs for Li–P–S materials and, perhaps even more importantly, the benchmarking of existing and new models. We have shown examples of how LiPS-25 can enable insights into the nature and applicability of graph-based foundation MLIPs, as well as into fine-tuning strategies.

Looking forward, physically grounded benchmarks like those presented here can serve as a general template for validating MLIPs. We have outlined protocols that span four levels of evaluation: starting with basic energetic validation (Task 1) and domain-specific force accuracy tests along relevant MD trajectories (Task 2) through to full dynamic property benchmarks, here, the ionic conductivity (Task 3), and finally to an assessment of robustness under a wide range of conditions, including very high temperatures and pressures (Task 4). Together, these tasks provide a structured framework for assessing MLIP quality that can guide model developers and users. While we have focused on the Li–P–S system in the present study, we expect that the framework (and associated code) can be readily adapted to other material systems of interest.

In the age of atomistic foundation models, systematic tests as outlined in this work could be incorporated into validation pipelines,^35^ community benchmarks,^36,37^ and automated MLIP development workflows.^121,122^ Embedding LiPS-25 and related benchmarks in this way would not only clarify how models behave across different regimes, but also guide their most effective use in downstream applications – ultimately supporting more reliable, transparent, and efficient use of MLIPs in computational materials chemistry.

## Supplementary Material



## Data Availability

Data supporting this work are available at https://github.com/nfragapane/lips-25.

## Notes

Additional details on data set construction and contents; computational details for benchmark tasks, model training and fine-tuning, and DFT calculations; and expanded reference experimental and computational ionic conductivity data for Li_7_P_3_S_11_ relevant to Task 3 (supplementary to Table 2) (PDF)
