# Supplementary material for: Li–P–S Electrolyte Materials as a Benchmark for Machine-Learned Interatomic Potentials
Source: J Chem Theory Comput. 2026 Mar 18;22(7):3646–59. doi: 10.1021/acs.jctc.5c02006 (PMC13085244; doi:10.1021/acs.jctc.5c02006)
Supplement: Supplementary file 1 [file ct5c02006_si_001.pdf]

Supporting Information for
"Li–P–S Electrolyte Materials as a Benchmark for Machine-Learned Interatomic Potentials"

Natascia L. Fragapane and Volker L. Deringer*

Inorganic Chemistry Laboratory, Department of Chemistry, University of Oxford, Oxford OX1 3QR, United Kingdom

E-mail: volker.deringer@chem.ox.ac.uk

S1 Dataset Construction

S1.1 Initial Dataset ("Iter0")

The LiPS-25 dataset is constructed to focus on the pseudo-binary Li_2S–P_2S_5 tie-line while maintaining broad coverage of the Li–P–S configurational space. The initial dataset (Iter0) and subsequent iterative melt–quench augmentations (Iter1-x, Iter2-x) were built around seven key compositions (see Figure 1a of the main text). The specific crystal structures used as starting points for AIMD annealing or melt–quench simulations are as follows: the constituent binary phases, Li_2S (anti-fluorite, $Fm\overline{3}m$; ICSD 60432), and P_2S_5 (a regular arrangement of P_4S_{10} molecules, $P\overline{1}$; ICSD 409061), as well as relevant ternary compounds, viz. $Li_2P_2S_6$ ($C2/m$; ICSD 253894), $Li_4P_2S_7$ ($P\overline{1}$; ref. S1), $Li_7P_3S_{11}$ ($P\overline{1}$; ICSD 157654), Li_3PS_4 ($Pmn2_1$; ICSD 180318), and Li_7PS_6 ($Pna2_1$; mp-1211324).

The Iter0 dataset comprises the following components, which are detailed below beyond the description given in the main text:

- *Crystalline*: All elemental (Li, P, S), binary, and ternary crystalline structures listed in the ICSD[S2] and Materials Project[S3] were included, with duplicates between the two databases removed and entries without full site occupancy excluded. For ternary crystals, both the primitive unit cells and $2 \times 2 \times 2$ supercells were considered. These structures underwent an initial DFT relaxation, followed by either a volume ($\pm 10\%$ around the relaxed volume) or angle (random angles within a range of 20% of relaxed cell angles) distortion, and atomic position "rattling" (with a standard deviation of 0.01 Å). These distortions aim to provide sampling around local minima of the potential energy surface.

- *AIMD snapshots*: For each of the seven key crystal structures, three separate 20 ps NVT AIMD runs were performed at 250, 500, and 1000 K, each at four densities scaled between the relaxed density and $2\times$ relaxed density. Every 1000-th frame was extracted and labeled.

- *Random Hard Sphere (RHS) Models*: Structures were generated using the `buildcell` code of *ab initio* random structure searching (AIRSS),[S4] with the latter code accessed using the Autoplex[S5] package. A minimum separation between atom pairs was enforced, defined as the average experimental crystalline values minus 0.5 Å.

- *Dimers*: Dimer configurations of all Li, P, and S pairs (viz. Li–Li, P–P, S–S, Li–S, Li–P, P–S) were sampled with interatomic separations of 1.0–2.0 Å in 0.1 Å intervals, and 2.0–7.0 Å with 0.2 Å intervals, within $20 \times 20 \times 20$ Å boxes. These configurations provide reference data for isolated pair interactions, including short-range repulsions and the onset of longer-range attractions.

Table S1: MQ protocols used in the iterative training procedure. The table reports the ensemble, temperature range for the melt-quench in the format "T_{start}-T_{melt}-T_{quench}", quench rate, and the type of structure extracted by the query-by-committee (QbC) procedure. The label "All" indicates that QbC had unrestricted selection of the most uncertain structures across compositions and trajectory points, including disordered crystalline, liquid, and glassy states. "Glasses only" indicates that QbC was restricted to the annealing stage following MQ, such that only amorphous structures were included.

**Iteration**	**Ensemble**	**MQ Temperatures (K)**	**Quench Rate (K/ps)**	**Structure type**
Iter1-1	NVT	300-1000-300	50	All
Iter1-2	NVT	300-1000-300	50	All
Iter1-3	NVT	300-1500-300	50	All
Iter1-4	NVT	300-1500-300	100	All
Iter2-1	NPT	300-1500-300	50	Glasses only
Iter2-2	NPT	300-1500-400	50	Glasses only
Iter2-3	NPT	300-1500-500	50	Glasses only

S1.2 Iterative Training ("Iter1" and "Iter2")

NequIP-driven[S6] melt–quench (MQ) simulations were employed to iteratively expand the Iter0 dataset, starting from the seven key compositions described above. At each iteration, the most uncertain structures were identified using a query-by-committee procedure: five subsampled models, each trained on a random 50% of the current dataset, were used to make predictions on a pool of evenly spaced snapshots collected from the MQ trajectories of all seven compositions. The 250 structures with the largest standard deviation in force predictions were then labeled and added to the dataset.

A timestep of 1 fs was used for all simulations. NVT runs employed a thermostat damping constant $t_{\mathrm{damp}}^{(T)} = 100$ fs, while NPT runs used $t_{\mathrm{damp}}^{(T)} = 10$ fs and a barostat damping constant $t_{\mathrm{damp}}^{(p)} = 100$ fs. Complete MQ simulation protocols for each iteration are provided in Table S1.

S2 Benchmark Tasks

S2.1 Task 1: Energetic accuracy

S2.1.1 Starting structures

For this task, the formation energies of eight relevant structures along the tie-line were calculated: $Li_2P_2S_6$ (ICSD 253894), $Li_4P_2S_7$ (ref. S1), $Li_7P_3S_{11}$ (ICSD 157654), α-Li_3PS_4 (ref. S7), β-Li_3PS_4 (mp-985583), γ-Li_3PS_4 (ICSD 180318), low-temperature Li_7PS_6 ($Pna2_1$, mp-1211324), and high-temperature Li_7PS_6 ($F\bar{4}3m$, ICSD 421130).

High-temperature Li_7PS_6 and β-Li_3PS_4 exhibit intrinsic disorder; for these, a single representative configuration was selected for benchmarking. Partial occupancies in high-temperature Li_7PS_6 were resolved using the `supercell` program,[S8] while the ordered structure provided by the Materials Project was used for β-Li_3PS_4 (mp-985583). The γ-Li_3PS_4 phase has near-full (0.99) S site occupancy, which was treated as fully occupied. All other structures are fully ordered.

For benchmarking, each representative structure was relaxed with either the MLIP or DFT, and the resulting formation energies were used for evaluation. This approach provides a consistent comparison of energetic accuracy, without aiming to capture the effects of full configurational disorder.

S2.1.2 Alternative calculation details and results

In the task as described in the main text, RMSE(E_f) is evaluated by relaxing each structure with either DFT or the MLIP model and then labeling with the same method – in line with the formal definition of E_f, but thereby conflating energetic and force accuracies. Here, we also provide the RMSE(E_f) computed from a fixed set of DFT-relaxed structures to isolate single-point energetic accuracy. These results are shown in Figure S1, and both protocols are implemented in the Jupyter notebook accompanying the present work.

S2.2 Task 2: Domain-specific force accuracy

S2.2.1 Simulation details

A melt–quench simulation of a 672-atom $Li_7P_3S_{11}$ supercell was performed in the NPT ensemble between 300 and 1500 K using a 1 fs timestep. The protocol consisted of a 25 ps annealing run at 300 K, a 50 ps melt ramp to 1500 K, a 25 ps anneal at 1500 K, and a 25 ps quench back to 300 K (corresponding to melt and quench rates of 24 K/ps),with damping parameters $t^{(T)}_{\mathrm{damp}} = 10$ fs and $t^{(p)}_{\mathrm{damp}} = 100$ fs. The simulation was driven by an interim NequIP potential from Iter2-2. DFT snapshots were extracted every 5 ps along the trajectory, and MLIP force errors were evaluated against these labels. The corresponding LAMMPS[S9] scripts and Jupyter Notebook for analysis are provided.

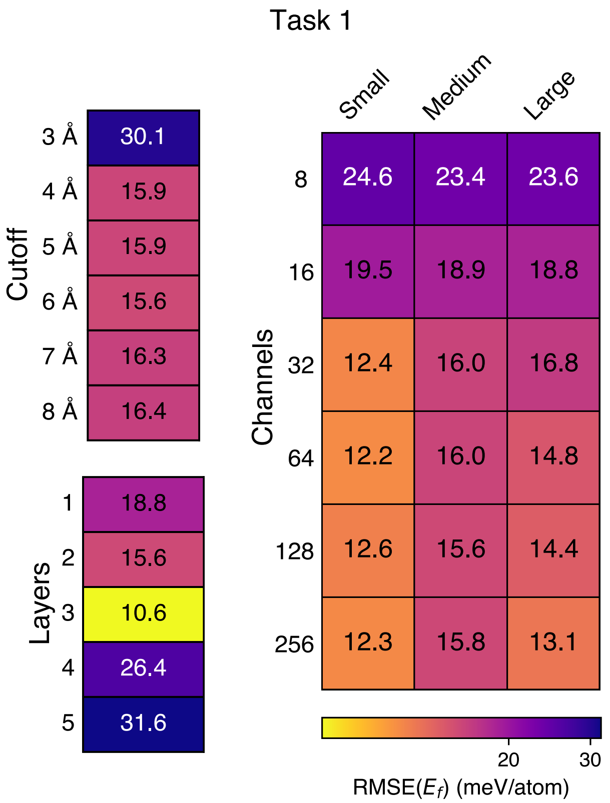

Figure S1: Task-1 performance for a MACE hyperparameter sweep. Errors are computed using the alternative protocol to the main text, i.e., from a fixed set of DFT-relaxed structures, and reported as the mean over five training repeats.

S2.3 Task 3: Property accuracy

S2.3.1 Simulation details

500 ps NVT anneals were performed on 672-atom $Li_7P_3S_{11}$ supercells at 400, 500, 667, and 800 K, with a timestep of 1 fs and $t^{(T)}_{\text{damp}} = 100$ fs. Example LAMMPS[S9] scripts are provided.

S2.3.2 Ionic conductivity calculation details

The Li-ion mean-square-displacement (MSD) is extracted from MD simulations using the MDAnalysis package,[S10,S11] according to:

$$\text{MSD}(t) = \frac{1}{N_{\text{Li}}} \sum_{i=1}^{N_{\text{Li}}} \left[\mathbf{R}_i(t) - \mathbf{R}_i(t=0)\right]^2 \tag{1}$$

where N_{Li} is the total number of Li ions, and $\mathbf{R}_i$ is the position of the i-th Li ion.

The diffusion coefficient (D_{Li}) at each temperature is extracted from the slope of a linear

fit to MSD vs t per block:

$$D_{\mathrm{Li}} = \frac{\mathrm{MSD}(t)}{2dt} \tag{2}$$

where d is the diffusion dimension ($d = 3$ here). Analysis of Li-ion motion is restricted to only the linear regime of MSD vs t, excluding the initial ballistic regime (the first 10 ps of the trajectory), and the block-averaging method (averaging D_{Li} over blocks of 20 ps) is used to extract a mean D_{Li} value.

The Arrhenius relation can be fitted to the temperature-dependent D values:

$$D_{\mathrm{Li}}(T) = D_0 \exp\left(\frac{-E_{\mathrm{a}}}{k_{\mathrm{B}}T}\right) \tag{3}$$

where D_0 and $\mathrm{E_a}$ refer to the pre-exponential factor and the activation energy, respectively, and the Boltzmann factor is given by k_{B}. These same values can be used to extrapolate to D at 298 K. To estimate the ionic conductivity at T=298 K, $\sigma_{298\mathrm{K}}$, the Nernst–Einstein relation is then used:

$$\sigma = \frac{N_{\mathrm{Li}} q^2 D_{\mathrm{Li}}(T)}{V k_{\mathrm{B}} T} \tag{4}$$

where V is the total volume of the simulated system, and q is the ionic charge of the Li^+ charge carriers (i.e., $q = e$). The accompanying Jupyter notebook for such trajectory analysis is provided.

To reduce computational expense, both the simulation time and supercell size were systematically converged (see Figure S2); from these tests, a 672-atom supercell and a simulation length of 500 ps simulations were deemed sufficient.

S2.3.3 Reference values for Task 3 (σ_{RT} of $Li_7P_3S_{11}$)

A comprehensive summary of reported experimental and computational values for the room-temperature ionic conductivity (σ_{RT}) of $Li_7P_3S_{11}$ is provided in Table S2. Experimental data were compiled from the review by Kudu et al.,[S12] where a detailed description of experimental synthesis conditions can be found, while computational references were collected independently in this work.

Experimental investigations on $Li_7P_3S_{11}$ have employed a variety of synthesis routes, including solid-state reactions, mechanochemical (ball-milling) methods, and wet-chemical techniques.[S12] However, direct comparison between experimental and computational values of σ_{RT} remains challenging for several reasons. Fully crystalline $Li_7P_3S_{11}$ is challenging to synthesize experimentally, meaning that samples usually contain a significant proportion of amorphous phase. The mixture of phases, and the resulting introduction of grain boundaries has been shown to strongly influence the ionic conductivity,[S13,S14] and is largely responsible for the wide range of reported experimental conductivities.

In contrast, computational studies typically simulate the intrinsic bulk conductivity of defect-free, fully crystalline $Li_7P_3S_{11}$ under periodic boundary conditions. Since these models

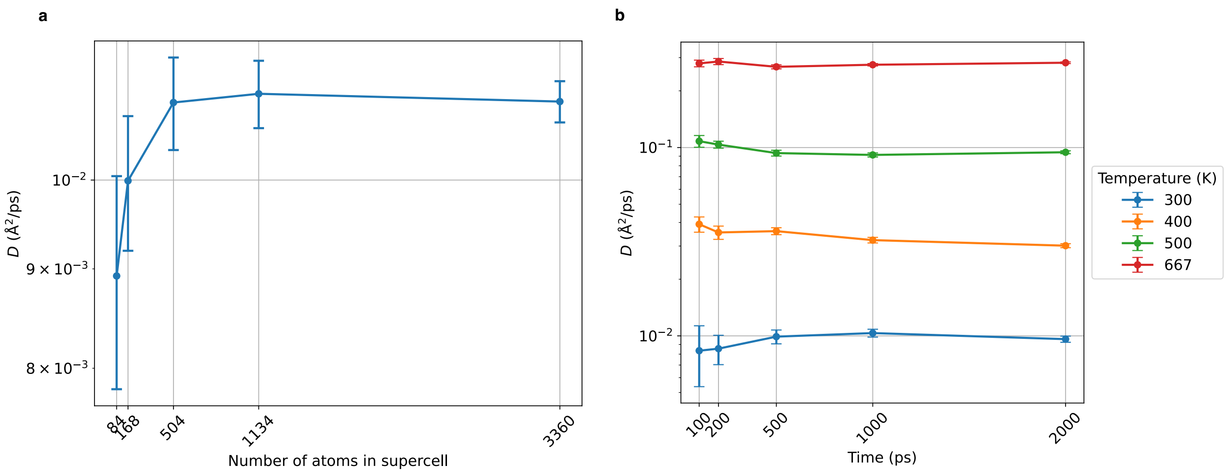

Figure S2: Convergence of predicted diffusion coefficients, D, with respect to (a) supercell size and (b) simulation length. In (a), values are obtained from 1 ns trajectories at 300 K for $Li_7P_3S_{11}$ supercells of increasing size. In (b), diffusion coefficients are evaluated for simulation lengths up to 1 ns at temperatures between 300 and 667 K using a 672-atom $Li_7P_3S_{11}$ supercell. Error bars represent the block-averaged standard error, i.e., the standard deviation of block diffusion coefficients divided by the square root of the number of blocks.

inherently neglect grain boundary effects and interfaces, direct comparison of experimental and computational values is of limited value. Additional sources of uncertainty in computational conductivity estimates arise from finite-size effects, limited simulation times, and the frequent use of the Nernst–Einstein relation to estimate σ_{RT} from diffusivity (see Section S2.3.1). Moreover, due to the high cost of AIMD, simulations are often performed with much lower convergence criteria than those typically used for DFT labels for training or fine-tuning, such as reduced plane-wave cutoffs, coarser k-point grids, and less strict electronic convergence thresholds. These compromises can introduce numerical noise or systematic errors in forces and energies, which may affect diffusion behavior and lead to deviations in predicted conductivities. Together, these factors contribute to systematic differences between experimental and theoretical σ_{RT} values; as such, quantitative agreement between experimental and computationally-derived conductivity data should not be expected.

For these reasons, we do not make direct comparisons between our MLIP-derived conductivities and experimental values. Instead, we consider predicted intrinsic bulk conductivities in the range of experiment to AIMD values to be reasonable, and as such, draw attention to ref. S15, reporting the highest known experimental conductivity, and ref. S16, reporting a representative AIMD value computed with the same exchange–correlation functional (PBEsol) as used in the present work.

Table S2: Room-temperature ionic conductivities (σ_{RT}) and activation energies (E_a) for $Li_7P_3S_{11}$ reported in the literature. For experimental studies, the synthesis method and the phase type obtained (glass, glass-ceramic, or crystalline) is indicated (collected from ref. S12). For computational studies, the simulation method and the phase modeled is noted.

**Ref.**	**Method**	**Phase**	σ_{RT} **(mS/cm)**	E_a **(eV)**
S15	Solid-state	Glass-ceramic	0.08	–
S15	Solid-state	Glass-ceramic	1.4	0.50
S15	Solid-state	Glass-ceramic	17	0.17
S16	Solid-state	Glass-ceramic	1.3	0.21
S16	Solid-state	Glass-ceramic	12	0.18
S17	Mechanochemical	Glass	0.04	0.41
S18	Mechanochemical	Glass	0.037	0.45
S19	Mechanochemical	Glass-ceramic	3.2	0.12
S20	Mechanochemical	Glass	0.05	0.38
S20	Mechanochemical	Crystal	4	0.29
S21	Mechanochemical	Glass	0.081	0.43
S21	Mechanochemical	Crystal	8.6	0.29
S22	Wet chemistry	Glass-ceramic	0.27	0.39
S23	Wet chemistry	Glass-ceramic	0.87	0.37
S24	Wet chemistry	Glass-ceramic	0.011	–
S24	Wet chemistry	Glass-ceramic	1.0	0.13
S16	AIMD (PBE)	Crystal	57.0	0.19
S16	AIMD (PBEsol)	Crystal	61.0	–
S25	AIMD (PBEsol)	Glass	0.082	–
S26	AIMD (PBE)	Crystal	45.7	0.19
S27	AIMD (PBE)	Crystal	72.0	0.17
S28	AIMD (PBE)	Crystal	84.0	0.17
S29	AIMD	Glass	1.8	–
S29	AIMD	Crystal	240.0	–

S2.4 Task 4: Robustness

A series of 100 ps NPT annealing runs was carried out for each MLIP model, across a 7×7 grid of (T, P) conditions spanning temperatures of 1000, 2000, 4000, 8000, 16 000, 32 000, and 64 000 K, and pressures of 10^6, 10^7, 10^8, 10^9, 10^{10}, 10^{11}, and 10^{12} Pa. Simulations used a 1 fs timestep, $t_{\mathrm{damp}}^{(T)} = 100$ fs, and $t_{\mathrm{damp}}^{(p)} = 1000$ fs. The starting point was an approximately cubic ($a \approx b \approx c$) 1008-atom random-hard-sphere $Li_7P_3S_{11}$ structure generated with the `buildcell` code,[S4] pre-relaxed in a fixed cell with the corresponding potential using the BFGS optimizer in ASE[S30] until $|f_{\max}| < 0.05$ eV/Å. Analysis scripts are provided for both success criteria: (i) simulation survival and (ii) the number of close-contact events.

S3 Experiments

S3.1 Benchmarking Graph-Based MLIPs

Benchmark results for Tasks 1 and 2 were obtained by averaging predictions from five models trained with different random seeds (see Section S3.4.4.). For Task 3, a single representative MACE model from these five was selected, and three sets of annealing runs were performed with different random seeds for initializing atomic velocities. The resulting σ_{298} predictions were then averaged across these three repeats.

S3.2 Fine-tuning Foundational Models with LiPS-25

To determine the optimal hyperparameters for the fine-tuning protocol described in Section S3.4.4, we varied the learning rate, the relative weighting of energy and force terms in the loss function, and the number of $Li_7P_3S_{11}$ structures in the fine-tuning dataset. Learning rates of 0.01, 0.001, and 0.0001 were tested: a setting of 0.01 led to significantly worse force predictions (particularly in the liquid regime), while 0.001 and 0.0001 performed comparably, with 0.0001 selected as optimal. Figure S3a shows the effect of dataset size, indicating that fine-tuning on 25 structures is sufficient, with little to no improvement from larger datasets. Figure S3b shows the effect of varying the energy:force weighting: increasing the force contribution gave negligible gains in force accuracy but substantially degraded energy predictions. Accordingly, a 1:1 weighting was adopted.

Each foundation model was fine-tuned with five different random seeds, and energy and force predictions from the resulting five models were averaged to produce the values shown in Figure 5.

S3.3 Ionic Conductivities from Fine-Tuned Models

S3.4 Computational Details

S3.4.1 DFT computations

For the construction of the LiPS-25 dataset, DFT reference computations were performed using VASP 6.4.3[S31–S34] with the PBEsol exchange–correlation functional[S35] and projector augmented-wave pseudopotentials (`PAW_PBE Li_sv 10Sep2004`, `PAW_PBE P 06Sep2000`, and `PAW_PBE S 06Sep2000`).[S36,S37] A plane-wave cutoff of 1000 eV and an energy tolerance of 10^{-8} eV per cell were chosen for SCF convergence. Brillouin-zone sampling was carried out using automatically generated k-point grids with a maximum spacing of 0.2 Å^{-1}.

For the initial structural optimization of crystalline structures in Iter0, the same plane-wave cutoff energy (1000 eV) was used, with an SCF energy tolerance of 10^{-6} eV per cell. The convergence criterion for ionic relaxation was a force tolerance of 10^{-2} eV Å^{-1}, and

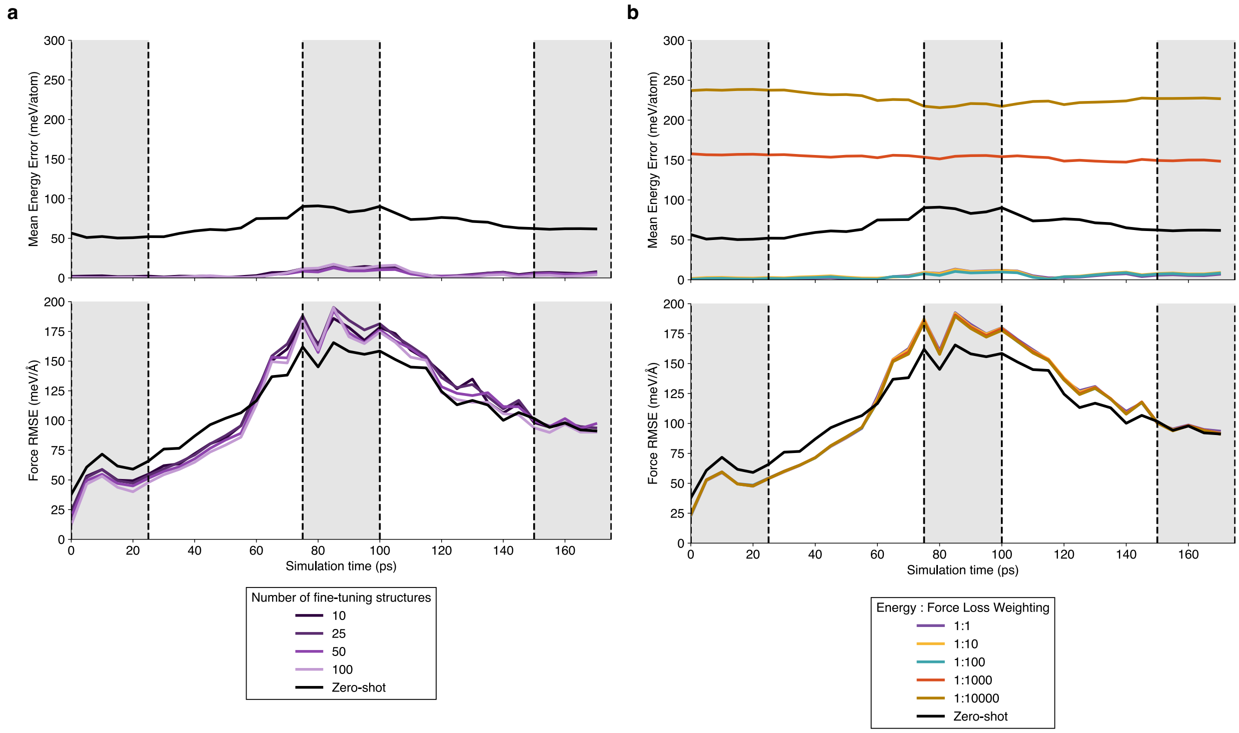

Figure S3: Hyperparameter optimization for the fine-tuning protocol, shown for MACE-OMAT-0 as a representative foundation model. Results are averaged over five repeats. (a) Effect of fine-tuning dataset size on energy and force errors: 25 $Li_7P_3S_{11}$ structures are sufficient, with little improvement from larger datasets. Tests were conducted using a 1:1 energy:force loss ratio. (b) Effect of varying the energy:force loss weighting: increasing the force contribution yields negligible improvements in force accuracy but substantially worsens energy predictions. Fine-tuning was performed on 25 structures.

reciprocal space was sampled using automatically generated k-point grids with a spacing of 0.2 $Å^{-1}$.

Ab initio molecular dynamics (AIMD) simulations were performed using VASP 6.3.2 to generate structures for the Iter0 dataset. These calculations employed a plane-wave cutoff of 400 eV, an SCF energy tolerance of 10^{-5} eV per cell, and Γ-point sampling only. The simulations were carried out in the NVT ensemble using a Nosé–Hoover thermostat, with a timestep of 1 fs.

S3.4.2 NequIP fitting

For dataset augmentation beyond Iter0, we employed NequIP.[S6] All models were trained on an NVIDIA RTX A6000 GPU in `float32` precision. The training hyperparameters were: cutoff radius $r_{\max} = 4.5$ Å; $l_{\max} = 2$; 32 features (including both even and odd); and 6 interaction layers. The invariant radial networks operated on a trainable Bessel basis of size 8 and were implemented with two hidden layers of 64 neurons, using SiLU nonlinearities.

Training used a learning rate of 0.001, a batch size of 50, and a loss function with equal weighting between energy and force terms. The learning rate was reduced by a factor of 0.5 if the validation loss did not improve for 100 epochs. Early stopping was applied if the validation loss failed to decrease by at least 0.005 over 40 epochs, otherwise the maximum number of epochs was set to 100,000. This model was then used to drive melt–quench simulations of the seven key compositions at each iteration.

At each iteration, a committee of five NequIP models with identical hyperparameters was trained on different random 50% subsets of the available dataset. This ensemble was used to identify and select the most uncertain MD frames for inclusion in the next training round.

S3.4.3 MACE fitting

For the hyperparameter sweep shown in Figure 3, five MACE models were trained with different random seeds for each set of hyperparameters. All models were trained on an NVIDIA A100 GPU using `float32` precision. Except for the cutoff sweep, a cutoff of 6 Å was used for all models along with the hyperparameters specified. Training was performed using the MACE implementation provided in `graph-pes`.[S38]

Hyperparameters not included in the sweep were kept at their default values as defined in `graph-pes`. Training was performed with a learning rate of 0.001 and a batch size of 32, except for the largest models where a reduced batch size of 5 was used due to memory constraints. The loss function combined energy and force terms in a 1:1 ratio. The learning rate was decreased by a factor of 0.8 if the validation loss did not improve over 25 epochs. Models were trained for a maximum of 1000 epochs.

S3.4.4 Fine-tuning

The foundation models used in this study were as follows: for the MACE family,[S39] MACE-MP-0b3, MACE-MPA-0, MACE-OMAT-0, and MACE-MATPES-0 (available at `https://github.com/ACEsuit/mace`); for the Orb family,[S40,S41] Orb-v2, Orb-v3-direct-inf-mpa, and Orb-v3-direct-inf-omat (available at `https://github.com/orbital-materials/orb-models`); and for the MatterSim family,[S42] MatterSim-1m and MatterSim-5m (available at `https://github.com/microsoft/mattersim`). Fine-tuning of the foundation models was carried out using the `graph-pes` package[S38] following a "naive" protocol, in which pre-trained weights are updated directly using the fine-tuning dataset. All models were trained on an NVIDIA RTX A6000 GPU in `float32` precision. A 6 Å cutoff with a learnable offset was used. Training employed a learning rate of 0.0001 and a batch size equal to the number of structures used for fine-tuning (25). The same loss function and learning rate schedule as described for MACE fitting in Section S3.4.3 were applied.

References

(S1) Holzwarth, N. A. W.; Lepley, N. D.; Du, Y. A. Computer modeling of lithium phosphate and thiophosphate electrolyte materials. *J. Power Sources* **2011**, *196*, 6870–6876.

(S2) Zagorac, D.; Müller, H.; Ruehl, S.; Zagorac, J.; Rehme, S. Recent developments in the Inorganic Crystal Structure Database: theoretical crystal structure data and related features. *J. Appl. Crystallogr.* **2019**, *52*, 918–925.

(S3) Jain, A.; Ong, S. P.; Hautier, G.; Chen, W.; Richards, W. D.; Dacek, S.; Cholia, S.; Gunter, D.; Skinner, D.; Ceder, G.; Persson, K. A. Commentary: The Materials Project: A materials genome approach to accelerating materials innovation. *APL Mater.* **2013**, *1*, 011002.

(S4) Pickard, C. J.; Needs, R. J. Ab initio random structure searching. *J. Phys.: Condens. Matter* **2011**, *23*, 053201.

(S5) Liu, Y.; Morrow, J. D.; Ertural, C.; Fragapane, N. L.; Gardner, J. L. A.; Naik, A. A.; Zhou, Y.; George, J.; Deringer, V. L. An automated framework for exploring and learning potential-energy surfaces. *Nat. Commun.* **2025**, *16*, 7666.

(S6) Batzner, S.; Musaelian, A.; Sun, L.; Geiger, M.; Mailoa, J. P.; Kornbluth, M.; Molinari, N.; Smidt, T. E.; Kozinsky, B. E(3)-equivariant graph neural networks for data-efficient and accurate interatomic potentials. *Nat. Commun.* **2022**, *13*, 2453.

(S7) Homma, K.; Yonemura, M.; Kobayashi, T.; Nagao, M.; Hirayama, M.; Kanno, R. Crystal structure and phase transitions of the lithium ionic conductor Li_3PS_4. *Solid State Ion.* **2011**, *182*, 53–58.

(S8) Okhotnikov, K.; Charpentier, T.; Cadars, S. Supercell program: a combinatorial structure-generation approach for the local-level modeling of atomic substitutions and partial occupancies in crystals. *J. Cheminform.* **2016**, *8*, 17.

(S9) Thompson, A. P.; Aktulga, H. M.; Berger, R.; Bolintineanu, D. S.; Brown, W. M.; Crozier, P. S.; in 't Veld, P. J.; Kohlmeyer, A.; Moore, S. G.; Nguyen, T. D.; Shan, R.; Stevens, M. J.; Tranchida, J.; Trott, C.; Plimpton, S. J. LAMMPS - a flexible simulation tool for particle-based materials modeling at the atomic, meso, and continuum scales. *Comput. Phys. Commun.* **2022**, *271*, 108171.

(S10) Michaud-Agrawal, N.; Denning, E. J.; Woolf, T. B.; Beckstein, O. MDAnalysis: A toolkit for the analysis of molecular dynamics simulations. *J. Comput. Chem.* **2011**, *32*, 2319–2327.

(S11) Gowers, R.; Linke, M.; Barnoud, J.; Reddy, T.; Melo, M.; Seyler, S.; Domański, J.; Dotson, D.; Buchoux, S.; Kenney, I.; Beckstein, O. MDAnalysis: A Python Package for the Rapid Analysis of Molecular Dynamics Simulations. Austin, Texas, 2016; pp 98–105.

(S12) Kudu, . U.; Famprikis, T.; Fleutot, B.; Braida, M.-D.; Le Mercier, T.; Islam, M. S.; Masquelier, C. A review of structural properties and synthesis methods of solid electrolyte materials in the $Li_2S–P_2S_5$ binary system. *J. Power Sources* **2018**, *407*, 31–43.

(S13) Minami, K.; Hayashi, A.; Tatsumisago, M. Crystallization Process for Superionic $Li_7P_3S_{11}$ Glass–Ceramic Electrolytes. *J. Am. Chem. Soc.* **2011**, *94*, 1779–1783.

(S14) Seino, Y.; Nakagawa, M.; Senga, M.; Higuchi, H.; Takada, K.; Sasaki, T. Analysis of the structure and degree of crystallisation of $70Li_2S–30P_2S_5$ glass ceramic. *J. Mater. Chem. A* **2015**, *3*, 2756–2761.

(S15) Seino, Y.; Ota, T.; Takada, K.; Hayashi, A.; Tatsumisago, M. A sulphide lithium super ion conductor is superior to liquid ion conductors for use in rechargeable batteries. *Energy Environ. Sci.* **2014**, *7*, 627–631.

(S16) Chu, I.-H.; Nguyen, H.; Hy, S.; Lin, Y.-C.; Wang, Z.; Xu, Z.; Deng, Z.; Meng, Y. S.; Ong, S. P. Insights into the Performance Limits of the $Li_7P_3S_{11}$ Superionic Conductor: A Combined First-Principles and Experimental Study. *ACS Appl. Mater. Interfaces* **2016**, *8*, 7843–7853.

(S17) Hayashi, A.; Hama, S.; Morimoto, H.; Tatsumisago, M.; Minami, T. Preparation of Li_2S-P_2S_5 Amorphous Solid Electrolytes by Mechanical Milling. *J. Am. Ceram. Soc.* **2004**, *84*, 477–79.

(S18) Dietrich, C.; Weber, D. A.; Sedlmaier, S. J.; Indris, S.; Culver, S. P.; Walter, D.; Janek, J.; Zeier, W. G. Lithium ion conductivity in $Li_2S–P_2S_5$ glasses – building units and local structure evolution during the crystallization of superionic conductors Li_3PS_4, $Li_7P_3S_{11}$ and $Li_4P_2S_7$. *J. Mater. Chem. A* **2017**, *5*, 18111–18119.

(S19) Mizuno, F.; Hayashi, A.; Tadanaga, K.; Tatsumisago, M. High lithium ion conducting glass-ceramics in the system $Li_2S–P_2S_5$. *Solid State Ion.* **2006**, *177*, 2721–2725.

(S20) Wenzel, S.; Weber, D. A.; Leichtweiss, T.; Busche, M. R.; Sann, J.; Janek, J. Interphase formation and degradation of charge transfer kinetics between a lithium metal anode and highly crystalline $Li_7P_3S_{11}$ solid electrolyte. *Solid State Ion.* **2016**, *286*, 24–33.

(S21) Busche, M. R.; Weber, D. A.; Schneider, Y.; Dietrich, C.; Wenzel, S.; Leichtweiss, T.; Schröder, D.; Zhang, W.; Weigand, H.; Walter, D.; Sedlmaier, S. J.; Houtarde, D.; Nazar, L. F.; Janek, J. *In Situ* Monitoring of Fast Li-Ion Conductor $Li_7P_3S_{11}$ Crystallization Inside a Hot-Press Setup. *Chem. Mater.* **2016**, *28*, 6152–6165.

(S22) Ito, S.; Nakakita, M.; Aihara, Y.; Uehara, T.; Machida, N. A synthesis of crystalline $Li_7P_3S_{11}$ solid electrolyte from 1,2-dimethoxyethane solvent. *J. Power Sources* **2014**, *271*, 342–345.

(S23) Wang, Y.; Lu, D.; Bowden, M.; El Khoury, P. Z.; Han, K. S.; Deng, Z. D.; Xiao, J.; Zhang, J.-G.; Liu, J. Mechanism of Formation of $Li_7P_3S_{11}$ Solid Electrolytes through Liquid Phase Synthesis. *Chem. Mater.* **2018**, *30*, 990–997.

(S24) Calpa, M.; Rosero-Navarro, N. C.; Miura, A.; Tadanaga, K. Preparation of sulfide solid electrolytes in the Li_2S–P_2S_5 system by a liquid phase process. *Inorg. Chem. Front.* **2018**, *5*, 501–508.

(S25) Baba, T.; Kawamura, Y. Structure and Ionic Conductivity of Li_2S–P_2S_5 Glass Electrolytes Simulated with First-Principles Molecular Dynamics. *Front. Energy Res.* **2016**, *4*.

(S26) Wang, Y.; Richards, W. D.; Bo, S.-H.; Miara, L. J.; Ceder, G. Computational Prediction and Evaluation of Solid-State Sodium Superionic Conductors $Na_7P_3X_{11}$ (X = O, S, Se). *Chem. Mater.* **2017**, *29*, 7475–7482.

(S27) Chang, D.; Oh, K.; Kim, S. J.; Kang, K. Super-Ionic Conduction in Solid-State $Li_7P_3S_{11}$-Type Sulfide Electrolytes. *Chem. Mater.* **2018**, *30*, 8764–8770.

(S28) Sadowski, M.; Albe, K. Computational study of crystalline and glassy lithium thiophosphates: Structure, thermodynamic stability and transport properties. *J. Power Sources* **2020**, *478*, 229041.

(S29) Ohkubo, T.; Ohara, K.; Tsuchida, E. Conduction Mechanism in $70Li_2S$-$30P_2S_5$ Glass by Ab Initio Molecular Dynamics Simulations: Comparison with $Li_7P_3S_{11}$ Crystal. *ACS Appl. Mater. Interfaces* **2020**, *12*, 25736–25747.

(S30) Hjorth Larsen, A.; Jørgen Mortensen, J.; Blomqvist, J.; Castelli, I. E.; Christensen, R.; Dułak, M.; Friis, J.; Groves, M. N.; Hammer, B.; Hargus, C.; Hermes, E. D.; Jennings, P. C.; Bjerre Jensen, P.; Kermode, J.; Kitchin, J. R.; Leonhard Kolsbjerg, E.; Kubal, J.; Kaasbjerg, K.; Lysgaard, S.; Bergmann Maronsson, J.; Maxson, T.; Olsen, T.; Pastewka, L.; Peterson, A.; Rostgaard, C.; Schiøtz, J.; Schütt, O.; Strange, M.; Thygesen, K. S.; Vegge, T.; Vilhelmsen, L.; Walter, M.; Zeng, Z.; Jacobsen, K. W. The atomic simulation environment—a Python library for working with atoms. *J. Phys.: Condens. Matter* **2017**, *29*, 273002.

(S31) Kresse, G.; Furthmüller, J. Efficient iterative schemes for ab initio total-energy calculations using a plane-wave basis set. *Phys. Rev. B* **1996**, *54*, 11169–11186.

(S32) Kresse, G.; Furthmüller, J. Efficiency of ab-initio total energy calculations for metals and semiconductors using a plane-wave basis set. *Comput. Mater. Sci.* **1996**, *6*, 15–50.

(S33) Kresse, G.; Hafner, J. Ab initio molecular-dynamics simulation of the liquid-metal–amorphous-semiconductor transition in germanium. *Phys. Rev. B* **1994**, *49*, 14251–14269.

(S34) Kresse, G. Ab initio molecular dynamics for liquid metals. *J. Non-Cryst. Solids* **1995**, *192-193*, 222–229.

(S35) Perdew, J. P.; Ruzsinszky, A.; Csonka, G. I.; Vydrov, O. A.; Scuseria, G. E.; Constantin, L. A.; Zhou, X.; Burke, K. Restoring the Density-Gradient Expansion for Exchange in Solids and Surfaces. *Phys. Rev. Lett.* **2008**, *100*, 136406.

(S36) Blöchl, P. E. Projector augmented-wave method. *Phys. Rev. B* **1994**, *50*, 17953–17979.

(S37) Kresse, G.; Joubert, D. From ultrasoft pseudopotentials to the projector augmented-wave method. *Phys. Rev. B* **1999**, *59*, 1758–1775.

(S38) Gardner, J. graph-pes: train and use graph-based ML models of potential energy surfaces. 2024; https://github.com/jla-gardner/graph-pes.

(S39) Batatia, I.; Benner, P.; Chiang, Y.; Elena, A. M.; Kovács, D. P.; Riebesell, J.; Advincula, X. R.; Asta, M.; Avaylon, M.; Baldwin, W. J.; Berger, F.; Bernstein, N.; Bhowmik, A.; Bigi, F.; Blau, S. M.; Cărare, V.; Ceriotti, M.; Chong, S.; Darby, J. P.; De, S.; Pia, F. D.; Deringer, V. L.; Elijošius, R.; El-Machachi, Z.; Falcioni, F.; Fako, E.; Ferrari, A. C.; Gardner, J. L. A.; Gawkowski, M. J.; Genreith-Schriever, A.; George, J.; Goodall, R. E. A.; Grandel, J.; Grey, C. P.; Grigorev, P.; Han, S.; Handley, W.; Heenen, H. H.; Hermansson, K.; Holm, C.; Ho, C. H.; Hofmann, S.; Jaafar, J.; Jakob, K. S.; Jung, H.; Kapil, V.; Kaplan, A. D.; Karimitari, N.; Kermode, J. R.; Kourtis, P.; Kroupa, N.; Kullgren, J.; Kuner, M. C.; Kuryla, D.; Liepuoniute, G.; Lin, C.; Margraf, J. T.; Magdău, I.-B.; Michaelides, A.; Moore, J. H.; Naik, A. A.; Niblett, S. P.; Norwood, S. W.; O'Neill, N.; Ortner, C.; Persson, K. A.; Reuter, K.; Rosen, A. S.; Rosset, L. A. M.; Schaaf, L. L.; Schran, C.; Shi, B. X.; Sivonxay, E.; Stenczel, T. K.; Svahn, V.; Sutton, C.; Swinburne, T. D.; Tilly, J.; Oord, C. v. d.; Vargas, S.; Varga-Umbrich, E.; Vegge, T.; Vondrák, M.; Wang, Y.; Witt, W. C.; Wolf, T.; Zills, F.; Csányi, G. A Foundation Model for Atomistic Materials Chemistry. *J. Chem. Phys.* **2025**, *163*, 184110.

(S40) Neumann, M.; Gin, J.; Rhodes, B.; Bennett, S.; Li, Z.; Choubisa, H.; Hussey, A.; Godwin, J. Orb: A Fast, Scalable Neural Network Potential. 2024; http://arxiv.org/abs/2410.22570.

(S41) Rhodes, B.; Vandenhaute, S.; Šimkus, V.; Gin, J.; Godwin, J.; Duignan, T.; Neumann, M. Orb-v3: atomistic simulation at scale. 2025; http://arxiv.org/abs/2504.06231.

(S42) Yang, H.; Hu, C.; Zhou, Y.; Liu, X.; Shi, Y.; Li, J.; Li, G.; Chen, Z.; Chen, S.; Zeni, C.; Horton, M.; Pinsler, R.; Fowler, A.; Zügner, D.; Xie, T.; Smith, J.; Sun, L.; Wang, Q.; Kong, L.; Liu, C.; Hao, H.; Lu, Z. MatterSim: A Deep Learning Atomistic Model Across Elements, Temperatures and Pressures. 2024; http://arxiv.org/abs/2405.04967.
